# Improved Sparrow Search Algorithm Based on Iterative Local Search

**DOI:** 10.1155/2021/6860503

**Published:** 2021-12-15

**Authors:** Shaoqiang Yan, Ping Yang, Donglin Zhu, Wanli Zheng, Fengxuan Wu

**Affiliations:** ^1^Xi'an Research Institute of High Technology, Xi'an, Shaanxi 710025, China; ^2^School of Information Engineering, Jiangxi University of Science and Technology, Ganzhou, Jiangxi 341000, China

## Abstract

This paper solves the shortcomings of sparrow search algorithm in poor utilization to the current individual and lack of effective search, improves its search performance, achieves good results on 23 basic benchmark functions and CEC 2017, and effectively improves the problem that the algorithm falls into local optimal solution and has low search accuracy. This paper proposes an improved sparrow search algorithm based on iterative local search (ISSA). In the global search phase of the followers, the variable helix factor is introduced, which makes full use of the individual's opposite solution about the origin, reduces the number of individuals beyond the boundary, and ensures the algorithm has a detailed and flexible search ability. In the local search phase of the followers, an improved iterative local search strategy is adopted to increase the search accuracy and prevent the omission of the optimal solution. By adding the dimension by dimension lens learning strategy to scouters, the search range is more flexible and helps jump out of the local optimal solution by changing the focusing ability of the lens and the dynamic boundary of each dimension. Finally, the boundary control is improved to effectively utilize the individuals beyond the boundary while retaining the randomness of the individuals. The ISSA is compared with PSO, SCA, GWO, WOA, MWOA, SSA, BSSA, CSSA, and LSSA on 23 basic functions to verify the optimization performance of the algorithm. In addition, in order to further verify the optimization performance of the algorithm when the optimal solution is not 0, the above algorithms are compared in CEC 2017 test function. The simulation results show that the ISSA has good universality. Finally, this paper applies ISSA to PID parameter tuning and robot path planning, and the results show that the algorithm has good practicability and effect.

## 1. Introduction

With the continuous emergence of various optimization problems, various algorithms and improved algorithms are emerging [1–4]. The emergence of swarm intelligence algorithm provides new ideas for solving various optimization problems. The swarm intelligence optimization algorithm is a meta-heuristic optimization algorithm that imitates the behavior of biological populations or biological behaviors and natural phenomena in the natural world. As the optimization effect of swarm intelligence algorithms is recognized by the public, swarm intelligence algorithms develop continuously, and more and more new swarm intelligence algorithms are proposed, such as firefly algorithm (FA) [5], ant lion optimizer (ALO) [6], whale optimization algorithm (WOA) [7], sine cosine algorithm (SCA) [8], crow search algorithm (CSA) [9], Harris hawks optimization algorithm (HHO) [10], slime mould algorithm (SMA) [11], hunger games search (HGS) [12], Runge–Kutta method (RUN) [13], and colony predation algorithm (CPA) [14].

Sparrow search algorithm (SSA) is a new group intelligence optimization algorithm proposed by Xue and Shen [15] in 2020. Inspired by sparrow foraging behavior, the algorithm has obvious advantages over traditional intelligent optimization algorithms, such as grey wolf Optimizer (GWO) [16], particle swarm optimization (PSO) [17], and genetic algorithm [18], with high stability, good search accuracy, and fast convergence [19]. Despite its fast convergence rate, the algorithm is prone to fall into local optimum and the results of optimization are random. To overcome this shortcoming, many scholars have proposed improved algorithms based on different strategies for the sparrow search algorithm and successfully solved many engineering problems. Based on the principle and model of sparrow search algorithm, Lv et al. proposed a fusion algorithm of bird swarm algorithm and sparrow search algorithm [20] and a chaotic sparrow search algorithm [21]. The former uses the search mechanism of bird swarm algorithm to add to the discoverers and followers of the sparrow search algorithm, which changes the update strategy of “full dimension reduction,” effectively breaks through the local restriction of search, and strengthens the global search ability; and the latter uses tent chaotic map to initialize the population, which makes the population more uniform. After one iteration, the second iteration of chaotic disturbance and Gaussian variation is carried out according to individual fitness and average fitness, which prevents local aggregation in the optimization process, enhances its ability to jump out of local optimization, and achieves good results in the application of image segmentation. A chaotic sparrow algorithm based on cubic mapping and elite reverse learning to initialize population is presented by Tang et al. [22]. At the same time, the sinusoidal algorithm is introduced, which balances the development and exploring ability of the algorithm. At the same time, when the algorithm comes to a standstill, the Gauss Walk strategy is used to jump out of the standstill, and its optimization performance is verified in 15 benchmark functions. Finally, the UAV track planning simulation is carried out in the case of threat. Compared with other optimization algorithms, the algorithm obtains the safe and feasible track with the best cost and meets the constraints. Ouyang et al. proposed a learning sparrow algorithm [23] adding lens reverse learning during the discoverer search phase makes the search more flexible and increases the diversity of the population. A spiral guidance mechanism is introduced to make the discoverer search more precise. Then, a local search mechanism is added to prevent the omission of high-quality solutions, and compared with other swarm intelligence algorithms in 12 basic test functions and CEC 2017 test sets, this shows good optimization ability. Finally, the improved sparrow search algorithm is validated in the robot path planning, and a stable and safe optimal path is planned.

The above algorithms have made some improvements on the basis of sparrow algorithm, but there are still some shortcomings:

1. There is still some randomness in the improved method of population initialization, which does not guarantee absolute uniformity of the population each time it is initialized.
2. The selected improved search strategy is subject to regional limitations, is easy to exceed the boundaries, and fails to perform effective global search in the whole space, resulting in a large number of individuals exceeding the boundaries and still trapped in local optimum.
3. By jumping directly to the discoverer, it is easy to miss the optimal solution. The improvement of local search accuracy is not significant, and there can be more improvement in search accuracy.
4. In terms of boundary control, the strategy of updating to boundary is adopted for individuals beyond the boundary, which does not make good use of the individual location and reduces the diversity of the population.

In order to solve the above problems, the sparrow search algorithm based on iterative local search is proposed in this paper. By variable helix factor and improved iterative local search, the effective utilization and search of individuals are improved. By adding a dimension by dimension lens learning strategy and change the focusing ability of the lens, the algorithm converges faster while helping to jump out of the local optimum, and improving the boundary strategy, the population diversity is increased. To verify the optimization performance of the algorithm, ISSA PSO, SCA, GWO [16], WOA, MWOA [24], SSA, BSSA, CSSA, and LSSA are tested and analyzed on 23 basic functions. To further verify the universality of the algorithm, the above algorithms are tested and analyzed in CEC 2017 test function [25,26]. Finally, ISSA is applied to PID parameter tuning [27]. The accuracy and convergence speed of the tuned results are improved compared with the SSA, which shows that the algorithm has good practicability. The main contributions of this paper are as follows:

1. In order to improve the effective use and search of individuals, a variable helix factor strategy is proposed and boundary control is improved
2. An improved iterative local search strategy is presented to improve the problem of low accuracy and missing better solutions in the search process
3. In order to improve the ability of the algorithm to jump out of local optimization, a dimension by dimension lens learning method is proposed to change the lens focusing ability
4. The versatility and flexibility of the algorithm using benchmark functions and CEC 2017 functions are validated
5. ISSA is used to optimize PID parameters to help quickly complete PID parameter tuning
6. ISSA is used to optimize the robot path planning problem and help get fast and stable results

The main work arrangement of this paper is as follows: Section 2 introduces the basic sparrow algorithm.  Section 3 introduces and analyses the ISSA.  Section 4 compares and analyzes the algorithm on the basic test function.  Section 5 compares and analyzes the algorithm on CEC 2017.  Section 6 applies the algorithm to PID parameter tuning.  Section 7 applies the algorithm to robot path planning.  Section 8 discusses and provides future research directions.

## 2. Sparrow Search Algorithm

In the process of sparrow foraging, there are two behavioral strategies: discoverer and follower. The individuals with better positions in the sparrow population generally take 10%–20% of the total population as discoverers, while the remaining individuals take part in the process. At the same time, 10%–20% of individuals are randomly assigned as scouters. The discoverer is responsible for leading the population in search direction and finding food, while the follower follows the discoverer to obtain food, and the scouter is alert to environmental threats and warns the sparrow population to move closer to a safe area.

In order to describe the process of sparrow foraging through mathematical models, it is necessary to formulate rules to simplify various behaviors of sparrows. The specific rules are as follows:

1. The individual energy of sparrow population depends on individual fitness evaluation, and the individual energy of discoverer is higher than that of discovers.
2. Once the scouters in the sparrow population find the threat of the external environment, they begin to send out an alarm signal. When the alert value is greater than the security threshold, the discoverers direct the population to the security zone.
3. Sparrows have flexible individual behavior strategies and can switch between discoverers and followers. As long as the individual energy reaches a certain degree, they can become discoverers, but the proportion between discoverers and followers in the population remains unchanged.
4. Sparrows with low energy may fly to other places for feeding in order to obtain higher energy.
5. When there is an external environmental threat, the sparrows at the edge of the population will quickly move to the safe area, and the sparrows in the middle of the population will immediately swim away to get close to other sparrows.

The discoverer is responsible for guiding the population to forage or to the location of the safe zone. The location update is described below:

(1) $$\begin{array}{c} X_{i,j}^{t+1}=\left\{\begin{array}{cc} X_{i,j}^{t}\cdot \;\exp\left(\frac{-i}{\alpha \cdot M}\right), & \text{if }R_{2}<\text{ST,}\\ X_{i,j}^{t}+Q\cdot L, & \text{if }R_{2}\geq \text{ST.} \end{array}\right. \end{array}$$

Among them, *t* represents the current number of iterations, and *M* is the maximum number of iterations. *X*_*i,j*_ represents the current position of the *i*-th sparrow in the *j*-th dimension. *α* ∈ [0, 1] and is a random number. *R*_2_ represents an early warning value, ST is the security threshold, and *R*_2_ ∈ [0, 1], ST ∈ [0.5, 1]. *Q* represents a random number that follows a normal distribution. *L* represents a 1 × *d* matrix with all elements 1. When *R*_2_ < ST, this indicates that the population environment is safe at this time, no predators are found around them, and the discoverers can conduct extensive searches to guide the population to higher energy levels. When *R*_2_ ≥ ST, this indicates that an individual within a population has discovered a predator and issued an alert, and that the discoverer quickly adjusts the search strategy to flee the current location, leading the population to a safe location.

In order to obtain high-quality food, followers follow the discoverer or forage alone, so the location of followers is updated as follows:

(2) $$\begin{array}{c} X_{i,j}^{t+1}=\left\{\begin{array}{cc} Q\cdot \;\exp\left(\frac{X_{\text{worst}}^{t}-X_{i,j}^{t}}{i^{2}}\right), & \text{if }i>\frac{n}{2},\\ X_{P}^{t+1}+\left|X_{i,j}^{t}-X_{P}^{t+1}\right|\cdot A^{+}\cdot L, & \text{otherwise.} \end{array}\right. \end{array}$$

Among them, *X*_*p*_ is the best position currently occupied by the discoverer, and *X*_worst_ represents the worst position currently. *A* is a 1 × *d* matrix with only 1 or −1 elements, where *A*^+^ = *A*^*T*^ (*AA*^*T*^)^−1^. When *i* > *n*/2, this indicates that the less adaptable 1st participant is not getting food, is very hungry, and needs to fly elsewhere to get more energy; when *i* < *n*/2, followers monitor the finder and compete for food with the finder with a higher predator, thereby increasing their energy.

When aware of the danger, the sparrow population will make antipredation behavior, and its mathematical expression is as follows

(3) $$\begin{array}{c} X_{i,j}^{t+1}=\left\{\begin{array}{cc} X_{\text{best}}^{t}+\beta \cdot \left|X_{i,j}^{t}-X_{\text{best}}^{t}\right|, & \text{if }f_{i}\neq f_{g},\\ X_{i,j}^{t}+K\cdot \left(\frac{\left|X_{i,j}^{t}-X_{\text{worst}}^{t}\right|}{\left(f_{i}-f_{w}\right)+\varepsilon }\right), & \text{if }f_{i}=f_{g}, \end{array}\right. \end{array}$$

where *X*_best_ represents the current global optimal position. *β* is the control step parameter and is a normally distributed random number with a mean value of 0 and a variance of 1. *K* is a random number belonging to [−1, 1], which controls the direction of the sparrow's movement as well as the step. *f*_*i*_ represents the fitness value of the current sparrow individual. *f*_*g*_ and *f*_*w*_ are the current optimal and worst fitness values, respectively. *ε* is a very small real number that prevents the denominator from being zero. When *f*_*i*_ ≠ *f*_*g*_, this indicates that the current sparrow is at the edge of the population and that an individual is vulnerable to predators, and it is necessary to approach other individuals in the population center to reduce the risk of predation. When *f*_*i*_ = *f*_*g*_, this indicates that individuals in the center of the population are aware of the danger and need to flee from their current location in order to avoid it.

## 3. Sparrow Search Algorithm Based on Iterative Local Search (ISSA)

### 3.1. Variable Helix Factor

Followers occupy the majority of individuals in the population. When *i* < (*N*/2), they have a unique update mechanism that draw closer to the discoverers quickly; it results in a fast convergence rate. When *i* > (*N*/2), they have the ability to search globally. However, the global search ability is not strong, limited by the boundary of the search area, which tends to cause aggregation at the boundary in the early stage, resulting in loss of population diversity, easy to fall into the local extreme phenomenon, and poor ability to jump out of the local optimal.

**Figure 1:**
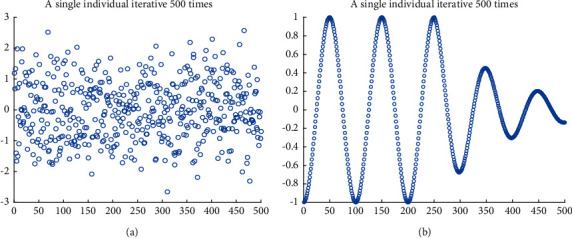
Coefficient model. (a) Original random coefficient. (b) Variable helix factor.

The location update method of follower adopts the random coefficient that obeys the normal distribution. Without considering the boundary, the coefficient has strong global search ability. However, when considering the boundary, this mechanism is detrimental to the individuals who are at or near the boundary. Many absolute coefficients exceed 1. When the boundary is exceeded, it causes the population individuals to aggregate at the boundary, does not make full use of the current location, and results in a significant decrease in population diversity and overall algorithm performance. Based on this, a variable helix factor is proposed to reduce the number of individuals beyond the boundary, control the search step and direction, make full use of the whole population space, the space for early search is large, maintain the diversity of the population, and help jump out of the trap of local optimum. Later local search is more detailed, which greatly improves the search ability of the algorithm, as shown in Figure 1.

The formula for the variable helix factor works as follows:

(4) $$\begin{array}{c} H=a\cdot \text{cos}\left(k\cdot l\cdot \pi \right), \end{array}$$

(5) $$\begin{array}{c} a=\left\{\begin{array}{cc} 1, & t<\frac{M}{2},\\ e^{5\cdot l}, & \text{otherwise}, \end{array}\right. \end{array}$$

(6) $$\begin{array}{c} l=1-2\cdot \frac{t}{M}. \end{array}$$

*H* is a variable helix factor, *a* is a parameter used to control the helix, with a value of 1 in the earlier period and a decreasing number of iterations in the later period; *k* is a parameter representing the helix cycle, (*M*/10) in general; and *l* is a parameter that decreases linearly from 1 to −1 in terms of the number of iterations.

**Figure 2:**
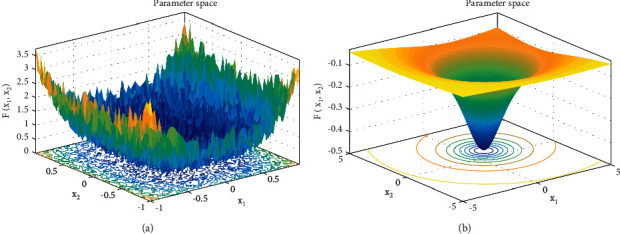
Parameter space. (a) Step function. (b) Shekel function.

In this paper, we select a high-dimensional test function, step function, and a low-dimensional test function, Shekel function, to test the original model and the improved model for individuals beyond the boundary, as shown in Figure 2. Set the population individual as 30 and the number of iterations as 500, run each for 10 times, and take the average value. The test results are shown in Table 1.

According to Table 1, when the followers conduct extensive search, the number beyond the boundary accounts for the vast majority of the total number beyond the boundary of the sparrow population, resulting in the loss of a large number of individuals. Therefore, it is necessary to restrict the extensive search scope of the followers; based on the variable helix factor, the number of times the followers exceeding the boundary is greatly reduced to 0, which fully retains the favorable position information of the current individual.

Improvements to the extensive search of the followers make it possible for the followers to make full use of the whole search space, get rid of the attraction of the local optimal solution more easily, strengthen the search for the whole space, maintain the diversity of the population, enhance the ability of algorithm exploration in the early stage, and enhance the ability of algorithm development in the later stage. Based on this, the formula is updated as follows:

(7) $$\begin{array}{c} x_{i,j}^{t+1}=\left\{\begin{array}{cc} \cos\left(a\cdot l\cdot \pi \right)\cdot \;\exp\left(\frac{x_{\text{worst}}^{t}-x_{i,j}^{t}}{i^{2}}\right), & i>\frac{N}{2}\text{ and }t<\frac{M}{2},\\ e^{5\cdot l}\cdot \;\cos\left(a\cdot l\cdot \pi \right)\cdot \;\exp\left(\frac{x_{\text{worst}}^{t}-x_{i,j}^{t}}{i^{2}}\right), & i>\frac{N}{2}\text{ and }t>\frac{M}{2}. \end{array}\right. \end{array}$$

### 3.2. Improved Iterative Local Search

When *i* < (*N*/2), the followers have a unique update mechanism that quickly closes to the discoverer's optimal solution, which results in fast convergence of the algorithm. The followers jump directly to the neighborhood of the discoverer's optimal solution. Although they have some development ability near the current optimal solution, they do not make enough use of the current solution and have poor stability. They cannot guarantee the quality and accuracy of the solution and have poor local development ability. Once trapped and unable to jump out of the local extreme state, the overall performance of the algorithm will be limited. Inspired by [28–30], this paper presents an improved iterative local search.

Local search algorithm [31] is a simple greedy search algorithm that is improved from the hill-climbing method. Local search starts from an initial solution, then searches the neighborhood of the solution, and updates the solution if there is a better solution or returns to the current solution. Iterative local search is an exploratory method that adds perturbation to the local optimal solution obtained by local search and then re-searches the local solution.

**Figure 3:**
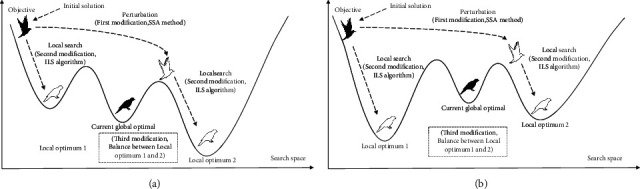
Main idea of improved iterative local search.

The improved iterative local search first performs a local search near the initial solution, then disturbs the initial solution by updating the location of the followers closer to the discoverer, and then searches the updated location again. It makes full use of the location information of the current individual and the location information of the current optimal solution to make the search more flexible. The first modification uses the mechanism by which the follower approaches the discoverer to disturb, so that the individual jumps near the current global optimal position to jump out of the local optimal position; the second modification uses the local search to obtain stable optimization results and improve the accuracy of the local search; and the third modification uses the local search result to obtain the optimal local search result to ensure the quality of the solution, as shown in Figure 3.

At the same time, two cases are illustrated in this paper.  Figure 3(a) indicates that after individual disturbance, a better solution can be found near the current global optimal solution; and Figure 3(b) indicates that an individual can find a better solution using his own favorable position, both of which help to jump out of the local optimal solution. This paper has not been replaced by the current global optimal solution to maintain population diversity and prevent premature convergence.

#### 3.2.1. The First Modification (SSA Method)

The improved iterative local search strategy is more suitable for this algorithm than the original iterative local search. It works by first disturbing the initial solution to get an intermediate solution (when *i* < *n*/2, the followers of the SSA are disturbed by a unique update mechanism that closes quickly to the finder's optimal solution). The initial and intermediate solutions are then searched again for a better solution. The first modification is used to solve the problem that the SSA is easy to fall into local optimal solution. The algorithm flow is as follows:

**Step 1 .**

The initial solution *X*^*∗*^ is perturbed by the current optimal solution *X*_best_ to obtain the intermediate solution *X*^*∗∗*^. The perturbation formula is as follows:

(8) $$\begin{array}{c} X^{**}=X_{\text{best}}+\left|X^{*}-X_{\text{best}}\right|\cdot A^{+}\cdot L. \end{array}$$

The formula for the original followers is as follows:

(9) $$\begin{array}{c} x_{i,j}^{t+1}=x_{P}^{t+1}+\left|x_{i,j}^{t}-x_{P}^{t+1}\right|\cdot A^{+}\cdot L,\;i<\frac{N}{2}, \end{array}$$

*X*_best_ is *x*_*P*_^*t*+1^, which is the best position the discoverer currently occupies; *X*^*∗*^ is *x*_*i*,*j*_^*t*^, which is the current position; *X*^*∗∗*^ is *x*_*i*,*j*_^*t*+1^, which is the updated location.

#### 3.2.2. The Second Modification (ILS Algorithm)

The second modification solves the problem of unstable and inaccurate optimization results of the SSA. The ILS algorithm here represents the local search stage in the ILS algorithm. It works by searching the initial and intermediate solutions locally to get a better solution and effectively utilizes the current position of the initial solution to prevent the individual from missing the better solution in the process of jumping directly to the current optimal solution. At the same time, local search near the current optimal solution helps to improve the accuracy of the solution and jump out of the local optimum in a small range. The algorithm flow is as follows:

**Step 2 .**

Initial solution *X*^*∗*^ starts local search, and the formula is as follows:

(10) $$\begin{array}{c} X^{1}=X^{*}\cdot \text{rand}\left(\;\right), \end{array}$$

rand( ) is a random number between 0 and 1.

**Step 3 .**

Intermediate solution *X*^*∗∗*^ starts local search, and the formula is as follows:

(11) $$\begin{array}{c} X^{2}=X^{**}\cdot \text{rand}\left(\;\right). \end{array}$$

#### 3.2.3. The Third Modification

The third modification is to optimize the local search results to ensure the quality of the solution. The working principle is to use greedy strategy to compare the local search results of the initial and intermediate solutions and select the better value as the final solution *X*. The algorithm flow is as follows:

**Step 4 .**

Calculate fitness *f*(*X*^1^) of *X*^1^.

**Step 5 .**

Calculate fitness *f*(*X*^2^) of *X*^2^

**Step 6 .**

Compare *f*(*X*^1^) and *f*(*X*^2^) to select the best individuals for location updates; that is,

  If *f*(*X*^1^) ≤ *f*(*X*^2^)
 
*X* = *X*^1^
  else *X* = *X*^2^
  end

Based on the SSA and the three modifications above, the formula of the follower (*i* < *N*/2) in the SSA is updated as follows:

(12) $$\begin{array}{c} x_{i,j}^{t+1}=\left\{\begin{array}{cc} x_{i,j}^{t}\cdot \text{rand}\left(\;\right), & i<\frac{N}{2}\text{ and }f\left(X^{1}\right)\leq f\left(X^{2}\right),\\ \left(x_{p}^{t+1}+\left|x_{i,j}^{t}-x_{p}^{t+1}\right|\cdot A^{+}\cdot L\right)\cdot \text{rand}\left(\;\right), & i<\frac{N}{2}\text{ and }f\left(X^{1}\right)>f\left(X^{2}\right). \end{array}\right. \end{array}$$

### 3.3. Dimension by Dimension Lens Imaging Learning

**Figure 4:**
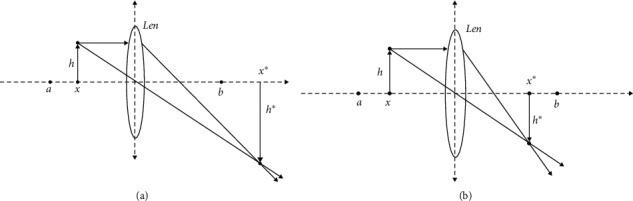
Main principles of lens imaging.

Swarm intelligence algorithms have the disadvantage of easily falling into local optimum. In this regard, relevant scholars have proposed the method of adding reverse learning to swarm intelligence algorithm [32–34]. The solution after reverse learning can be closer to the optimal solution. Generally, reverse learning can only search for the optimal solution in a certain space, but it still has monotonicity and the possibility of falling into local optimum. Lens learning [35, 36] has better optimization ability than general direction learning and can continuously converge to the optimal solution in a certain space. However, once there is no optimal solution in the selected space, it will still lead to local optimization in the end. At the same time, scouters have antipredator behavior to help the population to jump out of the local optimum, but their ability to jump out of the local optimum is unstable, resulting in sometimes unable to jump out of the local optimum. In view of this phenomenon, this paper proposes a dimension by dimension lens imaging learning strategy to change the focusing ability of the lens, which is used to strengthen the scouters' ability to jump out of the local optimum, lens learning for each dimension, and reduce the mutual interference between the dimensions. In the early stage, the lens with poor focusing ability is selected for reverse learning. At this time, the imaging is divergent and far away from the lens, which can help jump out of the local optimum, as shown in Figure 4(a); in the later stage, the lens with strong focusing ability is selected for reverse learning. The imaging is concentrated and close to the lens, which can accelerate the convergence, as shown in Figure 4(b). Comparison of three kinds of reverse learning is shown in Table 2.

The principle of lens imaging is as follows:

As shown in Figure 4, taking one-dimensional space as an example, it is assumed that there is an individual with a height of *h* at the position. Under the action of the lens, it forms an image with a height of *h*^*∗*^ at the position *x*^*∗*^. *a*, *b* is the boundary, and the lens position is the midpoint of [*a*, *b*]. According to the principle of lens imaging and triangle similarity principle:

(13) $$\begin{array}{c} \frac{\left(\left(a+b\right)/2\right)-x}{x^{*}-\left(\left(a+b\right)/2\right)}=\frac{h}{h^{*}}=k. \end{array}$$

By transforming the above formula, we can get

(14) $$\begin{array}{c} x^{*}=\frac{a+b}{2}+\frac{a+b}{2k}-\frac{x}{k}, \end{array}$$

*k* is used to indicate the focusing ability of the lens, that is, the imaging size. When *k* = 1, it can be simplified as follows:

(15) $$\begin{array}{c} x^{*}=a+b-x. \end{array}$$

This is the general reverse learning strategy. It can be seen that the general reverse learning strategy is a special case of lens imaging. The general reverse learning strategy *k* is fixed, and the obtained individuals are also fixed. Lens learning can change the position of individuals by adjusting *k*, so as to further enhance the diversity of groups. Generally, it takes a constant that is not equal to 1. This paper proposes a strategy of linearly increasing *K* according to the number of iterations, that is

(16) $$\begin{array}{c} k=a+\frac{t}{M}, \end{array}$$

where *a* is a small constant to prevent the previous iterative imaging from being too large, which is taken as 0.1 in this paper. The early *k* is small, and the imaging is large; in the later stage, *k* is near 1, and the imaging is slightly smaller, which can help convergence.

At the same time, lens imaging is extended to each dimension, and lens imaging reverse learning is performed for each dimension. The formula is extended as follows:

(17) $$\begin{array}{c} x_{j}^{*}=\frac{a_{j}+b_{j}}{2}+\frac{a_{j}+b_{j}}{2k}-\frac{x_{j}}{k}, \end{array}$$

where *j* is the current dimension, *a*_*j*_ is the lower bound of the *j*-th dimension, and *b*_*j*_ is the upper bound of the *j*-th dimension. At the same time, this paper adopts dynamic boundary:

(18) $$\begin{array}{c} a_{j}=\min\;\left(x_{j}\right),\\ b_{j}=\max\;\left(x_{j}\right), \end{array}$$

where min(*x*_*j*_) is the minimum value of the *j*-th dimension in all individuals, and max(*x*_*j*_) is the maximum value of the *j*-th dimension in all individuals. Because *a*_*j*_ and *b*_*j*_ do not represent the boundary of the whole search space, when the imaging exceeds the boundary [*a*_*j*_, *b*_*j*_], it may not exceed the boundary of the whole search space. Therefore, when *k* is small in the current period, the imaging will exceed the boundary of the current *j*-th dimension, which helps to expand the search range, reduces the possibility of premature stagnation in the early stage, and helps to jump out of the local optimum. Finally, the greedy strategy is adopted. If the fitness value of the reverse solution is small and better than the original solution, the solution is updated and applied to the algorithm as follows:

(19) $$\begin{array}{c} x_{i,j}^{t+1}=\left\{\begin{array}{cc} \frac{a_{j}^{t}+b_{j}^{t}}{2}+\frac{a_{j}^{t}+b_{j}^{t}}{2k}-\frac{x_{i,j}^{t+1}}{k}, & f\left(x^{*}\right)<f\left(x\right),\\ x_{i,j}^{t+1}, & \text{otherwise.} \end{array}\right. \end{array}$$

#### 3.3.1. Verify the Ability to Jump Out of Local Optimization

**Figure 5:**
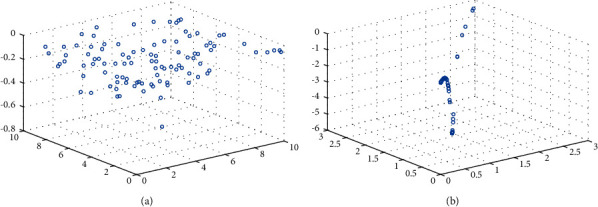
Individual distribution of SSA. (a) SSA individual initialization map. (b) Individual distribution of SSA in 20 generations.

**Figure 6:**
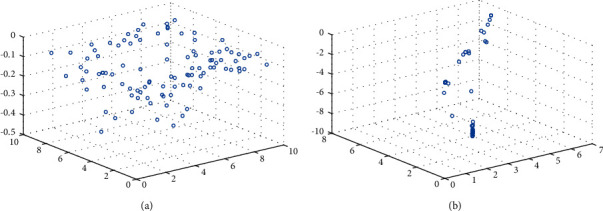
Individual distribution of ISSA. (a) ISSA individual initialization map. (b) Individual distribution of ISSA in 20 generations.

In this paper, the Shekel function mentioned above is selected as an example to draw the individual distribution diagram between the improved algorithm and the original algorithm to verify the ability of the above strategy to jump out of local optimization. The function image is shown in Figure 2. The selected population is 100, and the maximum number of iterations is 20. The individual distribution of the two algorithms are shown in Figures 5 and 6.

As can be seen from Figure 5, most individuals in the original algorithm have local aggregation and fall into local optimization. As can be seen from Figure 6, the improved algorithm has a larger search space, and most individuals converge near the optimal solution, effectively jumping out of the local optimal solution.

#### 3.3.2. Proof of Convergence of Dimension by Dimension Lens Imaging Learning

The proof of convergence of general refraction reverse learning swarm intelligence algorithm is given in reference [37]. Here, its conclusion is introduced to prove the convergence of the ISSA for dimensional lens back learning. It should be pointed out that the proof of convergence does not necessarily ensure that the algorithm converges to the global optimal solution. Since the SSA is also a swarm intelligence search algorithm, there are the following theorems:

**Theorem 1 .**

If the SSA algorithm based on general back learning converges, the ISSA algorithm also converges.

**Proof**

Let *x*_*i*_(*t*) and *x*_*i*_^*∗*^(*t*) be the current solution and reverse solution of generation *t*, *x*_*i*,*j*_(*t*) and *x*_*i*,*j*_^*∗*^(*t*) be the values of *x*_*i*_(*t*) and *x*_*i*_^*∗*^(*t*) in the *j*-th dimension, respectively, and the global optimal solution is *x*^best^. From Theorem 1,

(20) $$\begin{array}{c} \underset{t⟶\infty }{\lim}x_{i}\left(t\right)=x^{\text{best}}. \end{array}$$

Extended to the *j*-th dimension:

(21) $$\begin{array}{c} \underset{t⟶\infty }{\lim}x_{i,j}\left(t\right)=x_{j}^{\text{best}}. \end{array}$$

Due to *a*_*j*_(*t*)=min(*x*_*j*_(*t*)), *b*_*j*_(*t*)=max(*x*_*j*_(*t*)), we get

(22) $$\begin{array}{c} \underset{t⟶\infty }{\lim}a_{j}\left(t\right)=\underset{t⟶\infty }{\lim}b_{j}\left(t\right)=x_{j}^{\text{best}}. \end{array}$$

In the *t*-th generation, the current solution generated by the reverse learning strategy based on the lens imaging principle is

(23) $$\begin{array}{c} x_{i,j}^{*}\left(t\right)=\frac{a_{j}\left(t\right)+b_{j}\left(t\right)}{2}+\frac{a_{j}\left(t\right)+b_{j}\left(t\right)}{2k}-\frac{x_{i,j}\left(t\right)}{k}. \end{array}$$

When *t*⟶*∞*, from the above formula, we get

(24) $$\begin{array}{c} \underset{t⟶\infty }{\lim}x_{i,j}^{*}\left(t\right)=\underset{t⟶\infty }{\lim}\frac{a_{j}\left(t\right)+b_{j}\left(t\right)}{2}+\frac{a_{j}\left(t\right)+b_{j}\left(t\right)}{2k}-\frac{x_{i,j}\left(t\right)}{k}\\ =\underset{t⟶\infty }{\lim}\frac{x_{j}^{\text{best}}\left(t\right)+x_{j}^{\text{best}}\left(t\right)}{2}+\frac{x_{j}^{\text{best}}\left(t\right)+x_{j}^{\text{best}}\left(t\right)}{2k}-\frac{x_{j}^{\text{best}}}{k}=x_{j}^{\text{best}}. \end{array}$$

Return to the whole dimension:

(25) $$\begin{array}{c} \underset{t⟶\infty }{\lim}x_{i}^{*}\left(t\right)=x^{\text{best}}. \end{array}$$

It can be seen that when *x*_*i*_(*t*) converges to *x*^best^, the inverse solution *x*_*i*_^*∗*^(*t*) generated by lens learning also converges to *x*^best^. Therefore, if the SSA based on general reverse learning converges, the ISSA also converges.

### 3.4. Improved Boundary Control

In the standard SSA, when an individual in the optimization process exceeds the boundary, the boundary control will be carried out for the individual. The principle is as follows:

(26) $$\begin{array}{c} x_{i,j}^{t+1}=\left\{\begin{array}{cc} \text{Ub}, & x_{i,j}^{t+1}>\text{Ub},\\ \text{Lb,} & x_{i,j}^{t+1}<\text{Lb.} \end{array}\right. \end{array}$$

Ub and Lb are the upper and lower bounds of space, respectively. In this method, the strategy of turning the individuals beyond the boundary into the boundary will lead to the aggregation of individuals at the boundary and reduce the diversity of the population. It can be seen from Table 1 that although the improved strategy reduces the number of individuals beyond the boundary, some individuals still exceed the boundary and gather at the boundary. Literature [38] adopts the strategy of the current optimal solution for individuals beyond the boundary, which will be difficult to get rid of the local optimization. Therefore, this paper proposes a simple boundary treatment method, namely,

(27) $$\begin{array}{c} x_{i,j}^{t+1}=\text{Lb}+\left(\text{Ub}-\text{Lb}\right)\cdot \text{rand}\left(\;\right),\;x_{i,j}^{t+1}>\text{Ub and }x_{i,j}^{t+1}<\text{Lb}. \end{array}$$

In this method, the individuals beyond the boundary are randomly assigned to the search space, which makes more effective use of the population individuals and increases the diversity of the population more than that of the original algorithm.

### 3.5. Improved Sparrow Search Algorithm Flow

**Figure 7:**
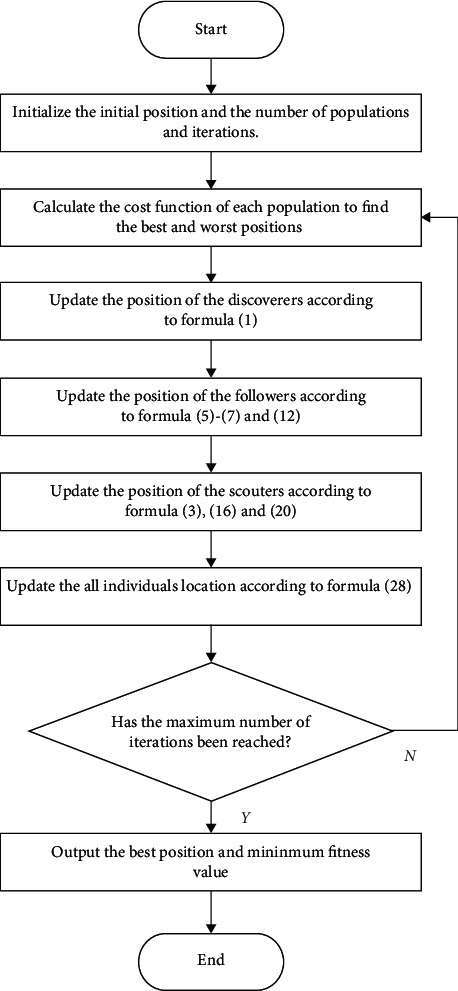
Algorithm flow chart.

In this paper, an improved sparrow search algorithm based on iterative local search is proposed. Firstly, the variable helix factor is used to improve the extensive search of followers, which reduces the individuals beyond the boundary and speeds up the convergence speed in the later stage. Secondly, the improved iterative local search is used to improve the local search of the followers. The initial solution is subject to local search and iterative local search after disturbance, which makes full use of the current position information to prevent premature convergence and improve the quality and accuracy of understanding. Then, the lens with changed focusing ability is used to carry out dimension by dimension lens imaging learning for the scouter, and it increases the search space and helps the population jump out of the local optimum. Finally, the boundary control strategy is improved to reduce the aggregation of individuals at the boundary and increase the diversity of the population. The introduction of various strategies makes the algorithm more flexible in the optimization ability, makes the population more diverse, strengthens its ability to get rid of local optimization, balances the global search ability and local search ability of the algorithm, and is conducive to finding reliable solutions. The specific algorithm flow is shown in Figure 7. The specific pseudo code is as follows:

### 3.6. Time Complexity Analysis

Time complexity is an important index to measure the performance of the algorithm, which is used to measure the running time of the algorithm. Assuming that the population size of the algorithm is *P*, the maximum number of iterations is *M* and the dimension is *D*, and the time complexity of the sparrow search algorithm is *O* (*P*·*M*·*D*). From a macro point of view, the improved sparrow search algorithm does not change the structure and cycle times of the algorithm, so its time complexity is also *O* (*P*·*M*·*D*), which is consistent with the original algorithm. From the microscopic point of view, the greedy strategy is adopted for iterative local search and dimension by dimension lens learning, which increases the algorithm complexity of some followers and all scouters to a certain extent, but the introduction of the improved strategy does not improve the order of magnitude of the algorithm, so the time complexity is still *O* (*P*·*M*·*D*).

## 4. Benchmark Function Test

In order to better verify the performance of ISSA, this paper selects 23 common basic test functions for verification and tests and compares them with 10 algorithms including PSO, SCA, GWO, WOA, MWOA, SSA, BSSA, CSSA, and LSSA. The specific parameter settings are shown in Table 3, and the test function information is shown in Table 4. F1–F7 is the high-dimensional single-peak benchmark function, F8–F13 is the high-dimensional multipeak benchmark function, and F14–F23 is the low-dimensional multipeak benchmark function. F1–F13 is tested in 100 dimensions to verify the performance of the algorithm in higher dimensions. For the sake of fairness, the population size and maximum number of iterations of each algorithm are 30 and 500, respectively, and each algorithm is run independently for 30 times to calculate its best value (best), worst value (worst), average value (AVE), and standard deviation (STD), and the optimal value of each index is processed in bold. Finally, each algorithm will be ranked according to the average value of the algorithm in the function. When the average values of the two algorithms are equal, the standard deviation will be compared. For performance evaluation, simulations are performed on Windows 10 Matlab 2016a, AMD Ryzen 7 4800U with Radeon Graphics @1.80 GHz with 16 GB RAM.

ISSA ranks first in most functions, and its average ranking is better than that of other algorithms. It can be seen from Tables 5 and 6 that, compared with other algorithms, ISSA has found the theoretical optimal value except F7, F10, F12, F13, and F15 and has found the optimal value in all algorithms in the above five functions. It can be seen that the ISSA has good ability to find the optimal solution. Among them, only the ISSA in F5 finds the theoretical optimal value, and only SSA and ISSA in F6 find the optimal value. The optimization accuracy of ISSA in F12 and F13 is a large number of orders of magnitude higher than that of other algorithms. When the dimension increases to 100, the optimization performance of ISSA is still very stable, while PSO, SCA, WOA, GOA, and SSA have a great impact on the optimization ability. It is worth mentioning that in F6 and F12, ISSA is only better than SSA and SSA improved the algorithm in terms of optimal value, but other indicators are better than other swarm intelligence algorithms. In F1–F13 function, ISSA finds a better solution than or the same solution as SSA, indicating that ISSA does not reduce the optimization ability of the algorithm. In the fixed dimension F14–F23, ISSA algorithm has good stability and the ability to jump out of the local optimum. It can find the solution close to the theoretical optimum almost every time.

**Figure 8:**
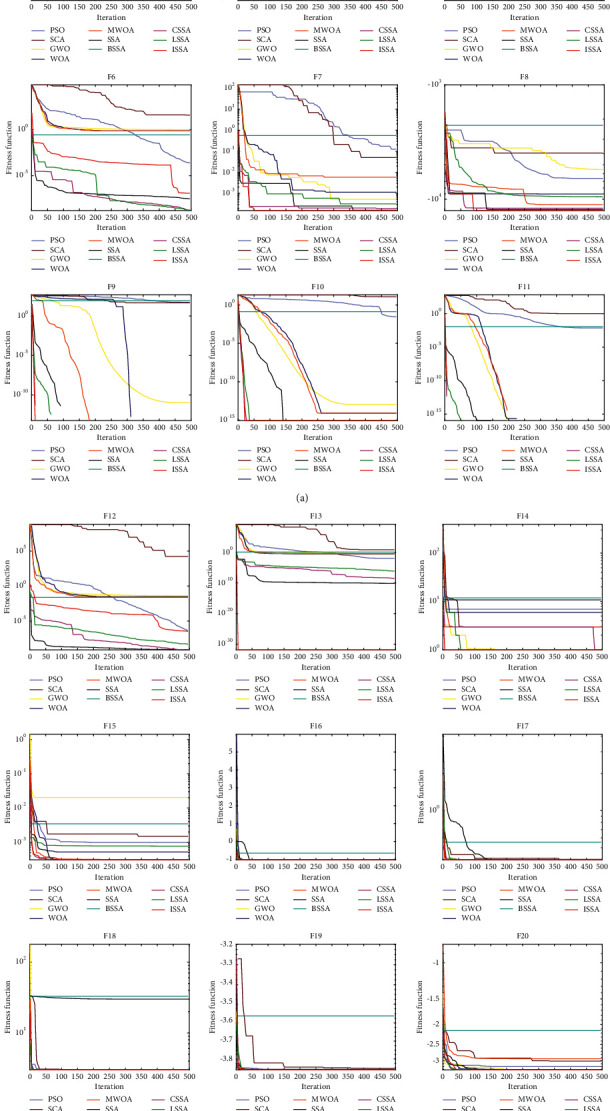
Convergence diagram of each algorithm.

In order to better describe the optimization ability, difference, and convergence speed of the algorithm with other algorithms, the Wilcoxon rank sum test results with other algorithms is given in Table 7 according to ISSA, and the convergence diagram of each algorithm is given in Figure 8. At the same time, in order to test the contribution of the four strategies to the algorithm, this paper selects a basic function to test the four components of the improved algorithm, as shown in the first figure in Figure 8. The above is based on the test results of 23 benchmark functions.

In this paper, the four strategies and the original SSA and ISSA are tested in the F1 function of the basic test function, as shown in the first figure in Figure 8. ISSA1, ISSA2, ISSA3, and ISSA4 represent the algorithms improved separately by the four improved strategies proposed before. Among them, ISSA2, ISSA3, and ISSA have found the theoretical optimal value. Compared with the original SSA, ISSA2 has significantly improved the convergence speed, reflecting its excellent search ability; ISSA3 suddenly converges to the theoretical optimal value in the middle of the iteration, indicating its excellent ability to jump out of the local optimal. Compared with the original SSA algorithm, the optimization results and convergence speed of ISSA1 and ISSA4 are improved by dozens of orders of magnitude, which can improve to a certain extent. It can be seen that improved iterative local search and dimension by dimension lens imaging learning play a more critical role in improving the ability of the algorithm. The combination of the above four strategies enables us to obtain an ISSA with faster convergence speed, more accurate results, and more stability.

In the Wilcoxon rank sum test, when the value is less than 0.05, it can be considered that there is a significant difference between the two. In Table 7, NaN indicates that their performance is equivalent and cannot be compared. It can be seen that most values are less than 0.05, indicating that the optimization performance of ISSA is significantly different from other algorithms, among which the difference between ISSA and CSSA is the smallest, followed by LSSA, BSSA, and SSA. As shown in Figure 8, the ISSA shows excellent optimization speed and convergence accuracy, and the convergence speed is fastest in most functions. The ISSA converges faster in the single-peak benchmark function and has better ability to resist the attraction local optimum in the multipeak benchmark function.

## 5. CEC 2017 Function Test

**Figure 9:**
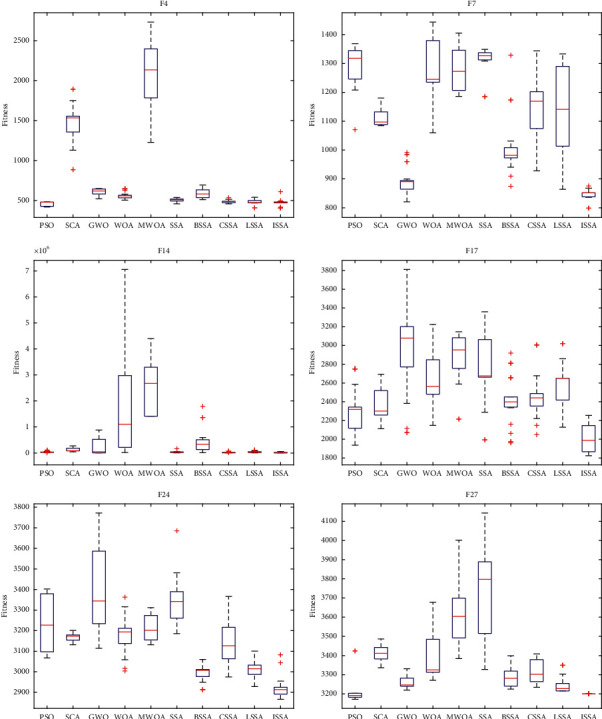
Box diagram of each algorithm.

In order to better illustrate the generality and effectiveness of the algorithm and avoid that the ISSA is only applicable to the case where the optimal value is 0, the algorithm is tested on the CEC 2017 test function. The evaluation times are 10000*∗*dim, the number of population individuals is 30, the dimension is 30, SD is set to 0.6, and other parameters remain unchanged. In this paper, the above algorithms are run independently for 30 times, and five indexes of each algorithm are calculated according to the results, namely, the best value (Best), the worst value (Worst), the median (Med), the average value (Ave), and the standard deviation (Std). Finally, each algorithm will be ranked according to the average value of the algorithm in the function. The optimal value of each index is treated in bold. Due to the defects of F2 function, it will not be tested in this paper. The specific test results are shown in Table 8. At the same time, six functions F4, F7, F14, F17, F24, and F27 are selected to draw the box diagram of the results, as shown in Figure 9.

It can be seen from the data in Table 8 that, in the 29 functions, ISSA ranks first in most functions and its average ranking is better than other algorithms. ISSA has good optimization effect and can be close to the theoretical optimal value of each function. In F1, F3, F6, F7, F8, F13,F14, F18, and F29, each index of ISSA is the best in the algorithm. Among the 20 functions, ISSA finds the optimal value of all algorithms, which shows that ISSA has strong optimization ability. When the optimal solution is not 0, ISSA shows better optimization performance than SSA, and PSO and CSSA also show good performance.

As can be seen from the box diagram in Figure 9, the ISSA has strong search ability and is closest to the theoretical optimal value among all algorithms. And ISSA's box graph has shorter length and stronger stability than other algorithms. Among them, the realization of SSA is poor because the individual in the SSA is directly jumping when approaching the current optimal solution, rather than moving to the current optimal solution like PSO. This problem leads to the rapid convergence of SSA, but it is easy to miss the high-quality solution and fall into local optimization. The ISSA uses the improved strategy to make up for this disadvantage, makes full use of the current solution, ensures the convergence speed, and increases its ability to jump out of the local optimum. But overall, the ISSA has better optimization performance than other algorithms, has good universality and effectiveness, and can adapt to some complex optimization problems.

## 6. PID Parameter Tuning

**Figure 10:**
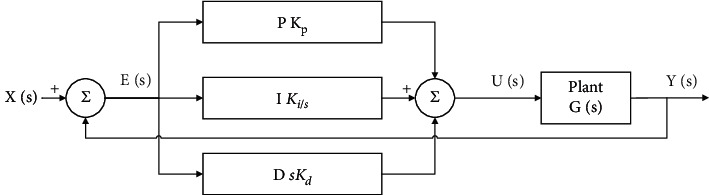
PID controller structure diagram.

A PID controller is the most widely used controller in the industry (accounting for about 90% of the controller). The PID controller is composed of three basic gain parameters to control the controlled object. It is mainly applicable to the system whose basic linear and dynamic characteristics do not change with time. Its structure is shown in Figure 10. When the proportional parameter *K*_*p*  _ increases, the rise time and steady-state error decrease. When the integral parameter *K*_*i*_ increases, the rise time is smaller, but the stability time and overshoot increase. The negative effect of the *K*_*i*_ increase can be overcome by adjusting the differential parameter *K*_*d*_. The relationship between output and input of the PID controller is as follows:

(28) $$\begin{array}{c} u\left(t\right)=K_{p}e\left(t\right)+K_{i}\int e\left(t\right)+K_{d}\frac{\text{d}e\left(t\right)}{\text{d}t}. \end{array}$$

Manual PID parameter tuning is a time-consuming process. Generally, it is tried through the experience and skills of engineers and the intelligent algorithm can complete the parameter tuning in a short time. In order to verify the practicability of ISSA, this paper uses ISSA to optimize PID parameters, simulates under unit step response and sinusoidal input response respectively, and tests with SSA to prove its optimization performance.

In this paper, the objective function [39] is set as follows:

(29) $$\begin{array}{c} F=\int_{0}^{\infty }\left(w_{1}|e\left(t\right)|+w_{2}u^{2}\left(t\right)\right)\text{d}t, \end{array}$$

**Figure 11:**
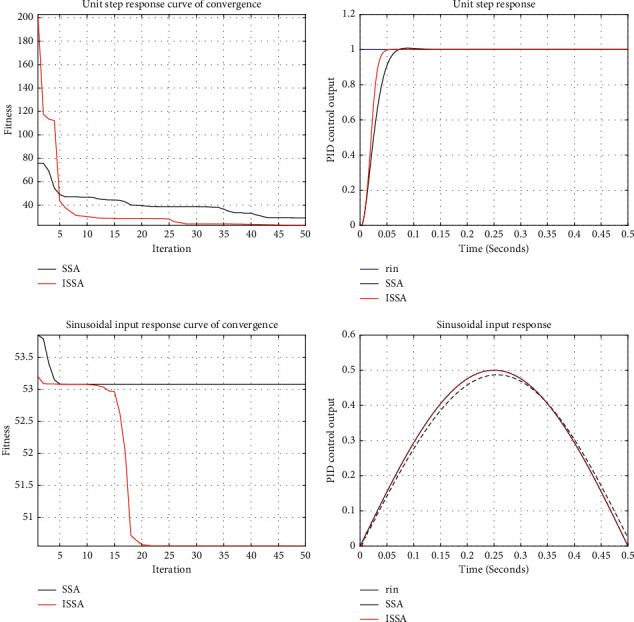
Convergence curve and PID control output.

*e*(*t*) is the error between the input value and the output value. Considering the dynamic characteristics of the iterative process, the integral of its absolute value is adopted; *u*(*t*) is the control value, which is added to avoid excessive control range; *w*_1_ and *w*_2_ are weights, and the value range is [0, 1]. In addition, measures shall be taken to prevent overshoot, that is, when overshoot occurs, an additional overshoot item shall be introduced into the objective function. At this time, the settings are as follows:

(30) $$\begin{array}{c} F=\int_{0}^{\infty }\left(w_{1}|e\left(t\right)|+w_{2}u^{2}\left(t\right)+w_{3}\left|e\left(t\right)\right|\right)\text{d}t,\;e\left(t\right)<0, \end{array}$$

where *w*_3_ is the weight and *w*_3_ ≫ *w*_1_. Generally, *w*_1_=0.999, *w*_2_=0.001, *w*_3_=100. Therefore, the goal of ISSA is to find a set of PID parameters to minimize the error of objective function. In this paper, the number of individuals and the number of iterations of the population are set to be 30 and 50, respectively. Other parameters are consistent with those of Table 2. The test is carried out under the condition of unit leap forward and sinusoidal input respectively and run independently for 10 times. The test results are shown in Figure 11, and the optimization results are shown in Table 9.

Since the results of 10 independent runs are consistent, only one is shown here. It can be seen from Figure 11 and Table 9 that in the unit step response, ISSA can complete the parameter setting in a very short time, and the convergence speed and accuracy are better than SSA. In the sinusoidal input response, ISSA can almost coincide with rin, and the tracking effect is significant. The tracking effect of SSA is slightly inferior. At the same time, the convergence speed and accuracy of ISSA are slightly better than SSA. The above results verify that ISSA has good algorithm performance, can quickly and accurately complete the PID parameter tuning, and help the system have shorter response time, higher system control accuracy, and better robustness. So far, the practicability has been proved.

## 7. Robot Path Planning

In PID parameter tuning, the dimension of the practical application is low. Therefore, this paper selects the discrete problem of more complex path planning to further verify the practicability of ISSA and makes a comparative experiment with SSA. In path planning, each sparrow is a feasible path. The environment modeling adopts the grid method, and the obstacles at the equivalent position are calculated according to the grid value. Grid number 0 is defined as feasible area and 1 as obstacle area. Then, the robot can plan the path on the grid specified as 0, and dimension *D* is the column number of the grid map. The cost function of the path length of the *i*-th sparrow is shown in

(31) $$\begin{array}{c} f\left(x\right)=\sum_{j=1}^{D-1}\sqrt{{\left(x_{j+1}+x_{j}\right)}^{2}+{\left(y_{j+1}-y_{j}\right)}^{2}}. \end{array}$$

**Figure 12:**
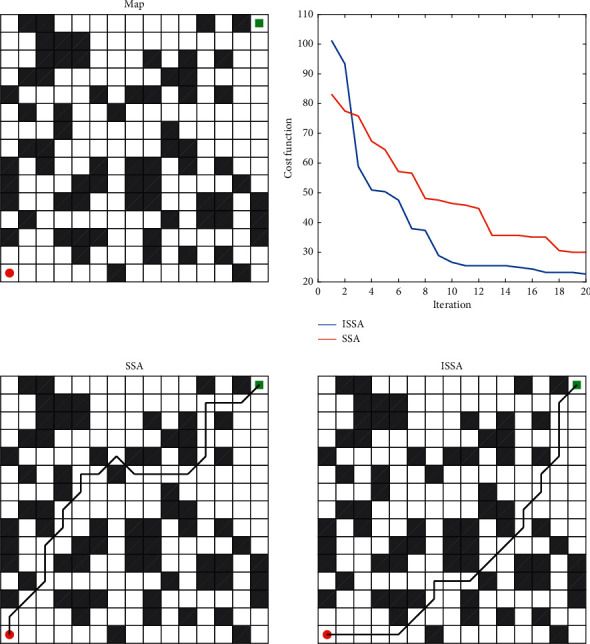
Convergence curve and optimal path.

In equation (31), *j* is the *j*-th dimension of a sparrow. Set each algorithm at 15 × 15, and the best path is shown in Figure 12. In order to eliminate the contingency, each algorithm is tested for 10 times, the optimal value, worst value, average value, and standard deviation of the fitness value of each algorithm are calculated, and these four indexes are used to measure the stability and feasibility of each algorithm. The optimization statistics of each algorithm are shown in Table 10.

It can be seen from the Table 10 and Figure 12, the minimum cost of ISSA planning is 19.7990, while the minimum cost of SSA is 22.6274. It can be seen that ISSA has strong path planning ability. According to other indicators, it can be seen that the path planned by ISSA has good stability. Therefore, ISSA has a good effect in more complex robot path planning and can plan a more stable and safer path.

## 8. Conclusions

This paper proposes an improved sparrow search algorithm based on iterative local search strategy, which introduces four strategies: variable helix factor, improved iterative local search, the lens imaging with changing focusing ability, and improved boundary control. ISSA overcomes the shortcomings of poor utilization of current individuals and lack of effective search and effectively improves the problems of falling into local optimal solution and low optimization accuracy. The test function results show that ISSA has good optimization performance and universality. The results of PID parameter tuning and robot path planning show that ISSA algorithm has good practicability.

The improved ISSA has good optimization performance, but it also has some shortcomings. For example, it can only find the optimal value in some functions, and other performance indicators are poor and unstable; a certain amount of work is added, resulting in a longer consumption time of the algorithm; and it did not improve the search scope of discoverers. In view of the shortcomings, we still need to do some work in the future: first, how to improve the stability of the algorithm; second, how to improve the search ability of followers; third, how to balance the time and optimization ability of the algorithm; and fourth, how to improve the search ability of discoverers on the basis of discoverers.

## Figures and Tables

**Algorithm 1:**
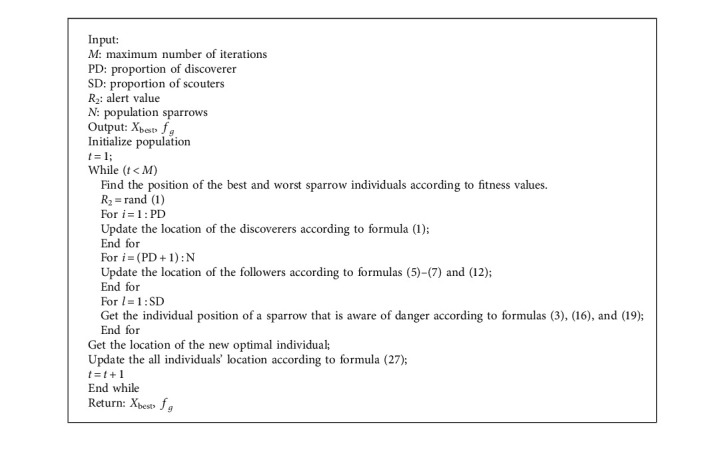
The framework of the ISSA.

**Table 1:** Statistics on the number of individuals beyond the boundary.

Function	Step	Shekel
Dimension	30 dim	4 dim
Boundary	$\left[\begin{array}{cc} -1.28 & 1.28 \end{array}\right]$	$\left[\begin{array}{cc} 0 & 10 \end{array}\right]$
Number of times the original algorithm follower exceeded the boundary	1505	3739
Number of times the follower exceeded boundaries after improvement	0	0
Total number of times the original algorithm exceeded the boundary	1696	4381
Total number of times the improved algorithm exceeded the boundary	161	1095

**Table 2:** Comparison of three kinds of reverse learning.

	Boundary	Focusing ability (*k*)	Reverse solution position	Effect
Reverse learning	Unchanged	1	On boundary midpoint symmetry	Accelerating convergence
Lens imaging learning	Dynamic change according to the maximum and minimum of individual position of population	A large constant, which is more than 1	About boundary midpoint reduction imaging	Accelerating convergence
Dimension by dimension lens imaging learning	The maximum and minimum values of each dimension change dynamically according to the individual position of the population	According to the dynamic change of iteration times, the first and middle stages are less than 1, and the later stage is more than 1	In the early and middle stages, the image is enlarged at the midpoint of the boundary, and in the later stage, the image is smaller	Jump out of the local optimum in the first and middle stage and accelerate the convergence in the later stage

**Table 3:** Parameter.

Algorithm	PSO	SCA	GWO	WOA	MWOA	SSA	BSSA	CSSA	LSSA	ISSA
Parameter	*c*1 = 2 *c*2 = 2 Wmix = 0.2 Wmax = 0.9	*a* = 2	*a* = (2 ⟶ 0)	*b* = 1	*b* = 1 *n* = 12000	ST = 0.8 PD = 0.2 SD = 0.2	ST = 0.8 PD = 0.2 SD = 0.2	ST = 0.8 PD = 0.2 SD = 0.2	ST = 0.8 PD = 0.2 SD = 0.2	ST = 0.8 PD = 0.2 SD = 0.2 *a* = 0.1

**Table 4:** Test function.

Function	Dimensions	Interval	Min
*F*_1_(*x*)=∑_*i*=1_^*n*^*x*_*i*_^2^	30/100	[−100, 100]	0
*F*_2_(*x*)=∑_*i*=1_^*n*^|*x*_*i*_|+∏_*i*=1_^*n*^|*x*_*i*_|	30/100	[−100, 100]	0
*F*_3_(*x*)=∑_*i*=1_^*n*^(∑_*j*=1_^*i*^*x*_*j*_)^2^	30/100	[−100, 100]	0
*F*_4_(*x*)=max_*i*_{|*x*_*i*_|, 1 ≤ *i* ≤ *n*}	30/100	[−100, 100]	0
*F*_5_(*x*)=∑_*i*=1_^*n*−1^[100(*x*_*i*+1_ − *x*_*i*_^2^)^2^+(*x*_*i*_ − 1)^2^]	30/100	[−30, 30]	0
*F*_6_(*x*)=∑_*i*=1_^*n*^([*x*_*i*_+0.5])^2^	30/100	[−100, 100]	0
*F*_7_(*x*)=∑_*i*=1_^*n*^*ix*_*i*_^4^+random[0,1)	30/100	[−1.28, 1.28]	0
$F_{8}\left(x\right)=\sum_{i=1}^{n}-x_{i}\sin\left(\sqrt{\left|x_{i}\right|}\right)$	30/100	[−500, 500]	−418.98*n*
*F*_9_(*x*)=∑_*i*=1_^*n*^[*x*_*i*_^2^ − 10 cos(2*πx*_*i*_)+10]	30/100	[−5.12, 5.12]	0
$F_{10}\left(x\right)=-20\;\exp\left(-0.2\sqrt{\left(1/n\right)\sum_{i=1}^{n}x_{i}^{2}}\right)-\exp\left[\left(1/n\right)\sum_{i=1}^{n}\cos\left(2\pi x_{i}\right)\right]+20+e$	30/100	[−32, 32]	0
$F_{11}\left(x\right)=\left(1/4000\right)\sum_{i=1}^{n}x_{i}^{2}-\prod_{i=1}^{n}\cos\left(x_{i}/\sqrt{i}\right)+1$	30/100	[−600, 600]	0
*F*_12_(*x*)=(*π*/*n*){10 sin(*πy*_1_)+∑_*i*=1_^*n*−1^(*y*_*i*_ − 1)^2^[1+10 sin^2^(*πy*_*i*+1_)]+(*y*_*n*_ − 1)^2^}+∑_*i*=1_^*n*^*u*(*x*_*i*_, 10,100,4)	30/100	[−50,50]	0
*y*_*i*_=1+((*x*_*i*_+1)/4)			
$u\left(x_{i},a,k,m\right)=\left\{\begin{array}{cc} k{\left(x_{i}-a\right)}^{m} & x_{i}>a\\ 0 & -a<x_{i}<a\\ k{\left(-x_{i}-a\right)}^{m} & x_{i}<-a \end{array}\right.$			
*F*_13_(*x*)=0.1{sin^2^(3*πx*_1_)+∑_*i*=1_^*n*^(*x*_*i*_ − 1)^2^[1+ sin^2^(3*πx*_*i*_+1)]+(*x*_*n*_ − 1)^2^[1+ sin^2^(2*πx*_*n*_)]}+∑_*i*=1_^*n*^*u*(*x*_*i*_, 5,100,4)	30/100	[−50,50]	0
*F*_14_(*x*)=((1/500)+∑_*j*=1_^25^1/(*j*+∑_*i*=1_^2^(*x*_*i*_ − *a*_*ij*_)^6^))^−1^	2	[−65.536, 65.536]	0.998
*F*_15_(*x*)=∑_*i*=1_^11^(*a*_*i*_ − (*x*_1_(*b*_*i*_^2^+*b*_1_*x*_2_)/(*b*_*i*_^2^+*b*_1_*x*_3_+*x*_4_)))^2^	4	[−5, 5]	0.0003
*F*_16_(*x*)=4*x*_1_^2^ − 2.1*x*_1_^4^+(1/3)*x*_1_^6^+*x*_1_*x*_2_ − 4*x*_2_^2^+4*x*_2_^4^	2	[−5, 5]	−1.032
*F*_17_(*x*)=(*x*_2_ − (5.1/4*π*^2^)*x*_1_ − 6)^2^+10(1 − (1/8*π*))cos *x*_1_+10	2	[−5, 5]	0.3979
*F*_18_(*x*)=[1+(*x*_1_+*x*_2_+1)^2^(19 − 14*x*_1_+3*x*_1_^2^ − 14*x*_2_+6*x*_1_*x*_2_+3*x*_2_^2^)] × [30+(2*x*_1_ − 3*x*_2_)^2^(18 − 32*x*_1_+12*x*_1_^2^+48*x*_2_ − 36*x*_1_*x*_2_+27*x*_2_^2^)]	2	[−2, 2]	3
*F*_19_(*x*)=∑_*i*=1_^4^*c*_*i*_exp(−∑_*j*=1_^3^*a*_*ij*_(*x*_*j*_ − *p*_*ij*_)^2^)	3	[0,1]	−3.863
*F*_20_(*x*)=∑_*i*=1_^4^*c*_*i*_exp(−∑_*j*=1_^6^*a*_*ij*_(*x*_*j*_ − *p*_*ij*_)^2^)	6	[0, 1]	−3.32
*F*_21_(*x*)=∑_*i*=1_^5^[(*X* − *a*_*i*_)(*X* − *a*_*i*_)^*T*^+*c*_*i*_]^−1^	4	[0, 10]	−10.1532
*F*_22_(*x*)=∑_*i*=1_^7^[(*X* − *a*_*i*_)(*X* − *a*_*i*_)^*T*^+*c*_*i*_]^−1^	4	[0, 10]	−10.4029
*F*_23_(*x*)=∑_*i*=1_^10^[(*X* − *a*_*i*_)(*X* − *a*_*i*_)^*T*^+*c*_*i*_]^−1^	4	[0, 10]	−10.5364

**Table 5:** Comparison table of the optimization effect of each algorithm (30 dimensions and fixed dimensions).

*F*	Index	PSO	SCA	GWO	WOA	MWOA	SSA	BSSA	CSSA	LSSA	ISSA
F1	Best	3.039*E* − 08	2.140*E* − 04	5.309*E* − 40	3.217*E* − 106	2.455*E* – 106	**0**	5.308*E* − 269	**0**	**0**	**0**
	Worst	2.030*E* − 06	1.076*E* + 01	9.250*E* − 38	3.331*E* − 94	1.392*E* − 95	**0**	9.853*E* − 249	**0**	**0**	**0**
	Ave	2.356*E* − 07	8.143*E* − 01	1.680*E* − 38	1.167*E* − 95	1.526*E* − 96	**0**	3.337*E* − 250	**0**	**0**	**0**
	Std	3.697*E* − 07	2.128*E* + 00	2.379*E* − 38	6.075*E* − 95	3.710*E* − 96	**0**	**0**	**0**	**0**	**0**
	Rank	9	10	8	7	6	1	5	1	1	1
F2	Best	5.760*E* − 05	4.632*E* − 05	3.354*E* − 23	2.808*E* − 63	1.441*E* − 62	**0**	1.827*E* − 136	**0**	2.123*E* − 263	**0**
	Worst	2.620*E* − 03	1.803*E* − 02	5.443*E* − 22	7.961*E* − 57	3.663*E* − 55	5.557*E* − 231	1.955*E* − 126	**0**	2.174*E* − 169	**0**
	Ave	4.306*E* − 04	4.924*E* − 03	1.410*E* − 22	1.014*E* − 57	1.594*E* − 56	2.250*E* − 232	1.332*E* − 127	**0**	7.253*E* − 171	**0**
	Std	4.897*E* − 04	4.613*E* − 03	1.008*E* − 22	2.073*E* − 57	6.709*E* − 56	**0**	4.462*E* − 127	**0**	**0**	**0**
	Rank	9	10	8	6	7	3	5	1	4	1
F3	Best	4.148*E* + 01	7.288*E* + 02	1.144*E* − 07	1.056*E* + 04	2.370*E* + 04	**0**	6.239*E* − 263	**0**	**0**	**0**
	Worst	1.465*E* + 02	1.962*E* + 04	3.338*E* − 03	5.602*E* + 04	6.525*E* + 04	6.949*E* − 59	3.651*E* − 232	**0**	1.751*E* − 244	**0**
	Ave	9.521*E* + 01	7.423*E* + 03	1.535*E* − 04	3.870*E* + 04	4.355*E* + 04	2.316*E* − 60	1.217*E* − 233	**0**	7.297*E* − 246	**0**
	Std	3.092*E* + 01	4.667*E* + 03	6.126*E* − 04	1.181*E* + 04	1.007*E* + 04	1.269*E* − 59	**0**	**0**	**0**	**0**
	Rank	7	8	6	9	10	5	4	1	3	1
F4	Best	6.129*E* − 01	1.282*E* + 01	9.960*E* − 08	3.426*E* + 00	4.515*E* − 02	2.102*E* − 293	3.783*E* − 130	**0**	1.942*E* − 210	**0**
	Worst	1.677*E* + 00	6.014*E* + 01	5.466*E* − 06	8.940*E* + 01	8.833*E* + 01	8.277*E* − 36	6.088*E* − 120	1.338*E* − 182	1.789*E* − 142	**0**
	Ave	1.077*E* + 00	3.790*E* + 01	1.135*E* − 06	4.785*E* + 01	4.205*E* + 01	2.774*E* − 37	4.300*E* − 121	4.720*E* − 184	6.026*E* − 144	**0**
	Std	2.676*E* − 01	1.281*E* + 01	1.271*E* − 06	2.867*E* + 01	3.029*E* + 01	1.511*E* − 36	1.332*E* − 120	**0**	3.265*E* − 143	**0**
	Rank	7	8	6	10	9	5	4	2	3	1
F5	Best	2.564*E* + 01	4.001*E* + 01	2.581*E* + 01	2.707*E* + 01	2.701*E* + 01	2.124*E* − 14	2.502*E* − 08	1.176*E* − 09	8.738*E* − 10	**0**
	Worst	1.817*E* + 02	2.485*E* + 05	2.874*E* + 01	2.877*E* + 01	2.876*E* + 01	3.498*E* − 05	2.572*E* − 04	5.266*E* − 04	1.097*E* − 03	**0**
	Ave	8.783*E* + 01	2.463*E* + 04	2.704*E* + 01	2.794*E* + 01	2.795*E* + 01	4.175*E* − 06	5.466*E* − 05	2.864*E* − 05	5.689*E* − 05	**0**
	Std	4.634*E* + 01	4.827*E* + 04	6.734*E* − 01	4.754*E* − 01	4.112*E* − 01	9.187*E* − 06	7.765*E* − 05	1.005*E* − 04	2.064*E* − 04	**0**
	Rank	9	10	6	7	8	2	4	3	5	1
F6	Best	7.636*E* − 06	5.178*E* + 00	6.012*E* − 07	4.835*E* − 02	6.880*E* − 02	**0**	3.524*E* − 11	1.360*E* − 12	3.385*E* − 10	**0**
	Worst	1.739*E* − 03	5.039*E* + 01	1.255*E* + 00	9.709*E* − 01	1.047*E* + 00	7.534*E* − 06	1.103*E* − 07	**5.188E − 08**	3.010*E* − 07	7.059*E* − 04
	Ave	2.531*E* − 04	1.420*E* + 01	7.218*E* − 01	4.287*E* − 01	3.361*E* − 01	3.019*E* − 07	1.788*E* − 08	**6.178E − 09**	3.970*E* − 08	1.409*E* − 04
	Std	3.327*E* − 04	1.305*E* + 01	3.225*E* − 01	2.176*E* − 01	2.303*E* − 01	1.372*E* − 06	2.881*E* − 08	**1.170E − 08**	7.458*E* − 08	1.965*E* − 04
	Rank	6	10	9	8	7	4	2	1	3	5
F7	Best	7.982*E* − 02	1.117*E* − 02	3.244*E* − 04	5.252*E* − 05	5.025*E* − 05	1.287*E* − 05	2.264*E* − 05	1.056*E* − 05	1.083*E* − 05	**8.231E − 06**
	Worst	3.788*E* − 01	2.485*E* − 01	4.318*E* − 03	1.108*E* − 02	1.460*E* − 02	1.062*E* − 03	9.727*E* − 04	**4.982E − 04**	8.427*E* − 04	5.115*E* − 04
	Ave	1.778*E* − 01	8.566*E* − 02	1.795*E* − 03	2.821*E* − 03	3.241*E* − 03	2.440*E* − 04	2.722*E* − 04	1.632*E* − 04	2.355*E* − 04	**1.506E − 04**
	Std	6.799*E* − 02	6.210*E* − 02	1.129*E* − 03	2.905*E* − 03	3.662*E* − 03	2.364*E* − 04	2.438*E* − 04	1.211*E* − 04	1.908*E* − 04	**1.196E − 04**
	Rank	10	9	6	7	8	4	5	2	3	1
F8	Best	−8.128*E* + 03	−4.963*E* + 03	−7.948*E* + 03	−1.257*E* + 04	−1.257*E* + 04	**−1.257E + 04**	−1.030*E* + 04	−1.257*E* + 04	−1.257*E* + 04	**−1.257E + 04**
	Worst	−3.967*E* + 03	−3.578*E* + 03	−3.642*E* + 03	−8.304*E* + 03	−7.837*E* + 03	−7.673*E* + 03	−7.671*E* + 03	−8.952*E* + 03	−1.033*E* + 04	**−1.083E + 04**
	Ave	−6.667*E* + 03	−4.023*E* + 03	−6.361*E* + 03	−1.153*E* + 04	−1.142*E* + 04	−1.060*E* + 04	−8.891*E* + 03	−1.128*E* + 04	−1.182*E* + 04	**−1.245E + 04**
	Std	9.060*E* + 02	3.243*E* + 02	7.713*E* + 02	1.520*E* + 03	1.230*E* + 03	1.948*E* + 03	5.289*E* + 02	8.299*E* + 02	6.529*E* + 02	**3.565E + 02**
	Rank	8	10	9	3	4	6	7	5	2	1
F9	Best	2.609*E* + 01	8.825*E* − 01	**0**	**0**	**0**	**0**	**0**	**0**	**0**	**0**
	Worst	8.661*E* + 01	2.056*E* + 02	1.045*E* + 01	**0**	1.137*E* − 13	**0**	**0**	**0**	**0**	**0**
	Ave	5.757*E* + 01	3.868*E* + 01	1.057*E* + 00	**0**	3.790*E* − 15	**0**	**0**	**0**	**0**	**0**
	Std	1.410*E* + 01	4.056*E* + 01	2.499*E* + 00	**0**	2.076*E* − 14	**0**	**0**	**0**	**0**	**0**
	Rank	10	9	8	1	7	1	1	1	1	1
F10	Best	2.946*E* − 03	2.093*E* − 02	7.905*E* − 14	**8.882E − 16**	**8.882E − 16**	**8.882E − 16**	**8.882E − 16**	**8.882E − 16**	**8.882E − 16**	**8.882E − 16**
	Worst	1.341*E* + 00	2.035*E* + 01	2.780*E* − 13	7.994*E* − 15	7.994*E* − 15	**8.882E − 16**	**8.882E − 16**	**8.882E − 16**	**8.882E − 16**	**8.882E − 16**
	Ave	2.508*E* − 01	1.104*E* + 01	1.554*E* − 13	3.849*E* − 15	5.033*E* − 15	**8.882E − 16**	**8.882E − 16**	**8.882E − 16**	**8.882E − 16**	**8.882E − 16**
	Std	4.770*E* − 01	9.347*E* + 00	4.636*E* − 14	2.483*E* − 15	2.653*E* − 15	**0**	**0**	**0**	**0**	**0**
	Rank	9	10	8	6	7	1	1	1	1	1
F11	Best	1.262*E* − 06	4.291*E* − 01	**0**	**0**	**0**	**0**	**0**	**0**	**0**	**0**
	Worst	3.694*E* − 02	2.192*E* + 00	2.243*E* − 02	2.284*E* − 01	**0**	**0**	**0**	**0**	**0**	**0**
	Ave	9.120*E* − 03	1.013*E* + 00	4.558*E* − 03	7.613*E* − 03	**0**	**0**	**0**	**0**	**0**	**0**
	Std	1.036*E* − 02	2.950*E* − 01	7.488*E* − 03	4.170*E* − 02	**0**	**0**	**0**	**0**	**0**	**0**
	Rank	9	10	7	8	1	1	1	1	1	1
F12	Best	4.722*E* − 08	1.373*E* + 00	6.907*E* − 03	4.668*E* − 03	2.905*E* − 03	5.172*E* − 19	3.481*E* − 13	4.456*E* − 13	2.676*E* − 09	**1.571E − 32**
	Worst	1.037*E* − 01	3.061*E* + 06	1.258*E* − 01	3.701*E* − 01	2.246*E* − 01	1.549*E* − 07	8.831*E* − 09	**3.088E − 09**	6.638*E* − 07	9.777*E* − 05
	Ave	6.914*E* − 03	1.092*E* + 05	4.088*E* − 02	3.888*E* − 02	2.754*E* − 02	7.364*E* − 09	8.349*E* − 10	**2.263E − 10**	1.373*E* − 07	2.558*E* − 05
	Std	2.630*E* − 02	5.587*E* + 05	2.534*E* − 02	6.678*E* − 02	3.888*E* − 02	2.905*E* − 08	1.843*E* − 09	**5.697E − 10**	1.673*E* − 07	3.011*E* − 05
	Rank	6	10	9	8	7	3	2	1	4	5
F13	Best	4.356*E* − 06	5.547*E* + 00	1.045*E* − 01	2.076*E* − 01	1.165*E* − 01	1.010*E* − 21	1.163*E* − 11	2.611*E* − 16	1.351*E* − 15	**1.350E − 32**
	Worst	1.152*E* − 02	3.410*E* + 06	9.183*E* − 01	1.069*E* + 00	1.303*E* + 00	2.333*E* − 07	2.900*E* − 07	1.083*E* − 10	3.592*E* − 11	**1.350E − 32**
	Ave	5.321*E* − 03	2.239*E* + 05	5.196*E* − 01	6.395*E* − 01	5.154*E* − 01	2.072*E* − 08	2.126*E* − 08	1.612*E* − 11	3.754*E* − 12	**1.350E − 32**
	Std	5.498*E* − 03	7.069*E* + 05	1.948*E* − 01	2.354*E* − 01	2.762*E* − 01	5.998*E* − 08	6.089*E* − 08	2.840*E* − 11	8.284*E* − 12	**0**
	Rank	6	10	8	9	7	4	5	3	2	1
F14	Best	**9.980E − 01**	**9.980E − 01**	**9.980E − 01**	**9.980E − 01**	**9.980E − 01**	**9.980E − 01**	**9.980E − 01**	**9.980E − 01**	**9.980E − 01**	**9.980E − 01**
	Worst	1.172*E* + 01	1.076*E* + 01	1.267*E* + 01	1.076*E* + 01	1.076*E* + 01	1.267*E* + 01	1.267*E* + 01	**9.980E − 01**	1.267*E* + 01	1.267*E* + 01
	Ave	4.147*E* + 00	2.251*E* + 00	4.065*E* + 00	2.767*E* + 00	3.907*E* + 00	8.622*E* + 00	1.086*E* + 01	**9.980E − 01**	1.585*E* + 00	1.453*E* + 00
	Std	3.028*E* + 00	1.888*E* + 00	4.163*E* + 00	2.953*E* + 00	3.791*E* + 00	5.438*E* + 00	4.143*E* + 00	**4.517E − 16**	2.179*E* + 00	2.149*E* + 00
	Rank	8	4	7	5	6	9	10	1	3	2
F15	Best	3.084*E* − 04	4.873*E* − 04	3.075*E* − 04	3.081*E* − 04	3.217*E* − 04	**3.075E − 04**	**3.075E − 04**	**3.075E − 04**	**3.075E − 04**	**3.075E − 04**
	Worst	1.093*E* − 03	1.506*E* − 03	2.036*E* − 02	5.941*E* − 03	2.252*E* − 03	3.447*E* − 04	6.256*E* − 04	3.205*E* − 04	**3.155E − 04**	3.319*E* − 04
	Ave	8.461*E* − 04	1.002*E* − 03	4.462*E* − 03	1.216*E* − 03	6.588*E* − 04	3.098*E* − 04	3.204*E* − 04	**3.079E − 04**	3.080*E* − 04	3.100*E* − 04
	Std	1.588*E* − 04	3.287*E* − 04	8.091*E* − 03	1.271*E* − 03	4.499*E* − 04	7.828*E* − 06	5.824*E* − 05	2.380*E* − 06	**1.552E − 06**	6.408*E* − 06
	Rank	7	8	10	9	6	3	5	1	2	4
F16	Best	**−1.032E + 00**	**−1.032E + 00**	**−1.032E + 00**	**−1.032E + 00**	**−1.032E + 00**	**−1.032E + 00**	**−1.032E + 00**	**−1.032E + 00**	**−1.032E + 00**	**−1.032E + 00**
	Worst	**−1.032E + 00**	**−1.031E + 00**	**−1.032E + 00**	**−1.032E + 00**	**−1.032E + 00**	−2.155*E* − 01	**−1.032E + 00**	**−1.032E + 00**	**−1.032E + 00**	**−1.032E + 00**
	Ave	**−1.032E + 00**	**−1.032E + 00**	**−1.032E + 00**	**−1.032E + 00**	**−1.032E + 00**	−1.004*E* + 00	**−1.032E + 00**	**−1.032E + 00**	**−1.032E + 00**	**−1.032E + 00**
	Std	**0**	7.279*E* − 05	**0**	**0**	**0**	1.490*E* − 01	**0**	**0**	**0**	**0**
	Rank	1	9	1	1	1	10	1	1	1	1
F17	Best	3.979*E* − 01	3.979*E* − 01	3.979*E* − 01	3.979*E* − 01	3.979*E* − 01	**3.979E − 01**	**3.979E − 01**	**3.979E − 01**	**3.979E − 01**	**3.979E − 01**
	Worst	3.979*E* − 01	4.108*E* − 01	3.985*E* − 01	3.980*E* − 01	3.980*E* − 01	**3.979E − 01**	**3.979E − 01**	**3.979E − 01**	**3.979E − 01**	**3.979E − 01**
	Ave	**3.979E − 01**	3.994*E* − 01	**3.979E − 01**	**3.979E − 01**	**3.979E − 01**	**3.979E − 01**	**3.979E − 01**	**3.979E − 01**	**3.979E − 01**	**3.979E − 01**
	Std	**0**	2.363*E* − 03	1.095*E* − 04	1.569*E* − 05	2.801*E* − 05	**0**	**0**	**0**	**0**	**0**
	Rank	1	10	9	7	8	1	1	1	1	1
F18	Best	**3.000E + 00**	**3.000E + 00**	**3.000E + 00**	**3.000E + 00**	**3.000E + 00**	**3.000E + 00**	**3.000E + 00**	**3.000E + 00**	**3.000E + 00**	**3.000E + 00**
	Worst	**3.000E + 00**	**3.000E + 00**	**3.000E + 00**	**3.000E + 00**	**3.000E + 00**	3.000*E* + 01	**3.000E + 00**	**3.000E + 00**	**3.000E + 00**	**3.000E + 00**
	Ave	**3.000E + 00**	**3.000E + 00**	**3.000E + 00**	**3.000E + 00**	**3.000E + 00**	4.800*E* + 00	**3.000E + 00**	**3.000E + 00**	**3.000E + 00**	**3.000E + 00**
	Std	**0**	**0**	**0**	**0**	**0**	6.850*E* + 00	**0**	**0**	**0**	**0**
	Rank	1	1	1	1	1	10	1	1	1	1
F19	Best	**−3.863E + 00**	−3.862*E* + 00	**−3.863E + 00**	**−3.863E + 00**	**−3.863E + 00**	**−3.863E + 00**	**−3.863E + 00**	**−3.863E + 00**	**−3.863E + 00**	**−3.863E + 00**
	Worst	**−3.863E + 00**	−3.854*E* + 00	−3.856*E* + 00	−3.861*E* + 00	−3.860*E* + 00	**−3.863E + 00**	**−3.863E + 00**	**−3.863E + 00**	**−3.863E + 00**	**−3.863E + 00**
	Ave	**−3.863E + 00**	−3.855*E* + 00	−3.862*E* + 00	−3.862*E* + 00	−3.862*E* + 00	**−3.863E + 00**	**−3.863E + 00**	**−3.863E + 00**	**−3.863E + 00**	**−3.863E + 00**
	Std	3.162*E* − 15	1.785*E* − 03	1.528*E* − 03	6.637*E* − 04	6.423*E* − 04	**0**	**0**	**0**	**0**	**0**
	Rank	6	10	7	8	9	1	1	1	1	1
F20	Best	**−3.322E + 00**	−3.224*E* + 00	**−3.322E + 00**	**−3.322E + 00**	**−3.322E + 00**	**−3.322E + 00**	**−3.322E + 00**	**−3.322E + 00**	**−3.322E + 00**	**−3.322E + 00**
	Worst	**−3.203E + 00**	−2.991*E* + 00	−3.138*E* + 00	−3.082*E* + 00	−3.087*E* + 00	**−3.203E + 00**	**−3.203E + 00**	**−3.203E + 00**	**−3.203E + 00**	**−3.203E + 00**
	Ave	−3.270*E* + 00	−3.078*E* + 00	−3.247*E* + 00	−3.258*E* + 00	−3.243*E* + 00	−3.263*E* + 00	−3.286*E* + 00	−3.263*E* + 00	−3.247*E* + 00	**−3.318E + 00**
	Std	5.993*E* − 02	6.050*E* − 02	6.344*E* − 02	7.915*E* − 02	6.898*E* − 02	6.047*E* − 02	5.542*E* − 02	6.047*E* − 02	5.828*E* − 02	**2.171E − 02**
	Rank	3	10	7	6	9	4	2	5	8	1
F21	Best	−1.015*E* + 01	−5.819*E* + 00	−1.015*E* + 01	−1.015*E* + 01	−1.015*E* + 01	−1.015*E* + 01	**−1.015E + 01**	**−1.015E + 01**	**−1.015E + 01**	**−1.015E + 01**
	Worst	−2.631*E* + 00	−4.973*E* − 01	−2.683*E* + 00	−4.984*E* + 00	−2.615*E* + 00	−5.055*E* + 00	−9.996*E* + 00	**−1.015E + 01**	**−1.015E + 01**	**−1.015E + 01**
	Ave	−7.630*E* + 00	−2.164*E* + 00	−9.088*E* + 00	−8.265*E* + 00	−8.515*E* + 00	−9.303*E* + 00	−1.013*E* + 01	**−1.015E + 01**	**−1.015E + 01**	**−1.015E + 01**
	Std	3.218*E* + 00	1.734*E* + 00	2.466*E* + 00	2.490*E* + 00	2.753*E* + 00	1.932*E* + 00	3.978*E* − 02	**0**	**0**	**0**
	Rank	9	10	6	8	7	5	4	1	1	1
F22	Best	**−6.207E + 00**	**−1.040E + 01**	**−1.040E + 01**	**−1.040E + 01**	**−1.040E + 01**	**−1.040E + 01**	**−1.040E + 01**	**−1.040E + 01**	**−1.040E + 01**	**−1.040E + 01**
	Worst	−9.028*E* − 01	−5.129*E* + 00	−1.838*E* + 00	−1.835*E* + 00	−5.088*E* + 00	−5.088*E* + 00	**−1.040E + 01**	**−1.040E + 01**	−5.088*E* + 00	**−1.040E + 01**
	Ave	−2.986*E* + 00	−1.023*E* + 01	−7.664*E* + 00	−7.782*E* + 00	−8.985*E* + 00	−9.850*E* + 00	**−1.040E + 01**	**−1.040E + 01**	−9.340*E* + 00	**−1.040E + 01**
	Std	1.774*E* + 00	9.629*E* − 01	3.007*E* + 00	3.125*E* + 00	2.391*E* + 00	1.615*E* + 00	**0**	**0**	2.162*E* + 00	**0**
	Rank	10	4	9	8	7	5	1	1	6	1
F23	Best	**−1.054E + 01**	−8.411*E* + 00	**−1.054E + 01**	**−1.054E + 01**	**−1.054E + 01**	**−1.054E + 01**	**−1.054E + 01**	**−1.054E + 01**	**−1.054E + 01**	**−1.054E + 01**
	Worst	−2.422*E* + 00	−9.439*E* − 01	−5.129*E* + 00	−2.422*E* + 00	−2.803*E* + 00	−5.129*E* + 00	−1.026*E* + 01	**−1.054E + 01**	**−1.054E + 01**	**−1.054E + 01**
	Ave	−9.395*E* + 00	−3.822*E* + 00	−1.018*E* + 01	−7.221*E* + 00	−8.252*E* + 00	−9.815*E* + 00	−1.051*E* + 01	**−1.054E + 01**	**−1.054E + 01**	**−1.054E + 01**
	Std	2.644*E* + 00	1.569*E* + 00	1.366*E* + 00	3.235*E* + 00	2.843*E* + 00	1.870*E* + 00	7.007*E* − 02	**0**	**0**	**0**
	Rank	7	10	5	9	8	6	4	1	1	1
Average rank	4.60	5.93	4.76	4.45	4.38	2.93	2.14	1.17	1.72	1.10	

**Table 6:** Comparison table of the optimization effect of each algorithm (100 dimensions).

*F*	Index	PSO	SCA	GWO	WOA	MWOA	SSA	BSSA	CSSA	LSSA	ISSA
F1	Best	9.872*E* + 00	9.308*E* + 02	2.693*E* − 12	4.175*E* − 83	2.894*E* − 84	**0**	2.714*E* − 255	**0**	**0**	**0**
	Worst	3.569*E* + 01	2.981*E* + 04	1.824*E* − 10	8.142*E* − 70	4.168*E* − 70	2.928*E* − 66	9.366*E* − 235	**0**	1.063*E* − 272	**0**
	Ave	2.206*E* + 01	1.131*E* + 04	4.086*E* − 11	4.068*E* − 71	1.464*E* − 71	1.010*E* − 67	3.248*E* − 236	**0**	3.935*E* − 274	**0**
	Std	5.407*E* + 00	7.239*E* + 03	4.038*E* − 11	1.574*E* − 70	7.601*E* − 71	5.438*E* − 67	**0**	**0**	**0**	**0**
	Rank	9	10	8	6	5	7	4	1	3	1
F2	Best	2.295*E* + 01	1.120*E* − 01	1.379*E* − 07	4.706*E* − 55	4.940*E* − 57	**0**	1.226*E* − 127	**0**	9.989*E* − 215	**0**
	Worst	5.920*E* + 01	2.205*E* + 01	5.637*E* − 07	3.020*E* − 47	1.360*E* − 48	8.218*E* − 35	1.609*E* − 115	**0**	4.528*E* − 140	**0**
	Ave	3.901*E* + 01	7.362*E* + 00	2.368*E* − 07	1.053*E* − 48	1.017*E* − 49	3.229*E* − 36	5.982*E* − 117	**0**	1.509*E* − 141	**0**
	Std	9.152*E* + 00	6.263*E* + 00	8.618*E* − 08	5.508*E* − 48	3.266*E* − 49	1.515*E* − 35	2.945*E* − 116	**0**	8.268*E* − 141	**0**
	Rank	10	9	8	6	5	7	4	1	3	1
F3	Best	1.196*E* + 04	1.540*E* + 05	2.370*E* + 01	4.152*E* + 05	5.261*E* + 05	**0**	9.631*E* − 256	**0**	**0**	**0**
	Worst	2.610*E* + 04	4.486*E* + 05	3.307*E* + 03	1.682*E* + 06	1.491*E* + 06	5.484*E* − 51	1.912*E* − 231	**0**	**0**	**0**
	Ave	1.660*E* + 04	2.370*E* + 05	1.037*E* + 03	1.007*E* + 06	1.040*E* + 06	1.906*E* − 52	6.898*E* − 233	**0**	**0**	**0**
	Std	3.793*E* + 03	6.543*E* + 04	8.578*E* + 02	3.146*E* + 05	2.624*E* + 05	1.018*E* − 51	**0**	**0**	**0**	**0**
	Rank	7	8	6	9	10	5	4	1	1	1
F4	Best	9.191*E* + 00	8.607*E* + 01	7.311*E* − 01	2.040*E* + 01	1.568*E* + 00	2.570*E* − 282	1.681*E* – 130	**0**	3.573*E* − 261	**0**
	Worst	1.493*E* + 01	9.584*E* + 01	8.765*E* + 00	9.697*E* + 01	9.638*E* + 01	7.474*E* − 32	4.501*E* – 118	**0**	4.421*E* − 158	**0**
	Ave	1.220*E* + 01	9.061*E* + 01	2.931*E* + 00	7.732*E* + 01	7.614*E* + 01	2.491*E* − 33	2.897*E* – 119	**0**	1.474*E* − 159	**0**
	Std	1.550*E* + 00	2.355*E* + 00	1.931*E* + 00	2.188*E* + 01	2.359*E* + 01	1.365*E* − 32	9.208*E* − 119	**0**	0	**0**
	Rank	7	10	6	9	8	5	4	1	3	1
F5	Best	7.371*E* + 03	2.331*E* + 07	9.657*E* + 01	9.745*E* + 01	9.752*E* + 01	1.531*E* − 14	1.382*E* − 07	2.260*E* − 08	8.400*E* − 10	**0**
	Worst	3.114*E* + 04	2.554*E* + 08	9.849*E* + 01	9.858*E* + 01	9.846*E* + 01	3.712*E* − 03	2.409*E* − 03	3.776*E* − 04	1.007*E* − 03	**0**
	Ave	1.571*E* + 04	1.182*E* + 08	9.778*E* + 01	9.819*E* + 01	9.814*E* + 01	1.501*E* − 04	3.859*E* − 04	8.782*E* − 05	2.173*E* − 04	**0**
	Std	5.206*E* + 03	6.138*E* + 07	5.923*E* − 01	2.454*E* − 01	2.609*E* − 01	6.750*E* − 04	6.669*E* − 04	1.081*E* − 04	2.744*E* − 04	**0**
	Rank	9	10	6	8	7	3	5	2	4	1
F6	Best	1.156*E* + 01	2.016*E* + 03	7.714*E* + 00	2.371*E* + 00	1.979*E* + 00	**0**	7.437*E* − 10	1.845*E* − 09	8.530*E* − 10	**0**
	Worst	3.269*E* + 01	3.267*E* + 04	1.146*E* + 01	8.063*E* + 00	6.289*E* + 00	**2.874E − 06**	1.995*E* − 05	4.954*E* − 06	1.287*E* − 04	7.775*E* − 03
	Ave	2.134*E* + 01	1.089*E* + 04	9.632*E* + 00	4.301*E* + 00	3.952*E* + 00	**1.845E − 07**	1.796*E* − 06	6.391*E* − 07	7.234*E* − 06	1.427*E* − 03
	Std	5.340*E* + 00	6.941*E* + 03	1.016*E* + 00	1.423*E* + 00	1.118*E* + 00	**5.339E − 07**	3.757*E* − 06	1.034*E* − 06	2.359*E* − 05	2.389*E* − 03
	Rank	9	10	8	7	6	1	3	2	4	5
F7	Best	1.198*E* + 03	4.974*E* + 01	2.106*E* − 03	2.143*E* − 04	1.476*E* − 04	2.729*E* − 05	9.448*E* − 06	1.484*E* − 05	9.049*E* − 06	**2.371E − 06**
	Worst	2.001*E* + 03	4.241*E* + 02	1.629*E* − 02	1.567*E* − 02	2.330*E* − 02	1.299*E* − 03	1.096*E* − 03	3.918*E* − 04	1.621*E* − 03	**3.207E − 04**
	Ave	1.518*E* + 03	1.609*E* + 02	7.634*E* − 03	4.709*E* − 03	5.183*E* − 03	3.217*E* − 04	3.569*E* − 04	1.502*E* − 04	4.565*E* − 04	**1.394E − 04**
	Std	1.889*E* + 02	9.512*E* + 01	3.085*E* − 03	5.310*E* − 03	5.977*E* − 03	2.954*E* − 04	2.842*E* − 04	1.087*E* − 04	4.189*E* − 04	**9.063E − 05**
	Rank	10	9	8	6	7	3	4	2	5	1
F8	Best	−1.781*E* + 04	−7.934*E* + 03	−1.999*E* + 04	−4.187*E* + 04	−4.190*E* + 04	**−4.190E + 04**	−2.698*E* + 04	**−4.190E + 04**	**−4.190E + 04**	**−4.190E + 04**
	Worst	−5.016*E* + 03	−6.076*E* + 03	−5.790*E* + 03	−2.378*E* + 04	−2.746*E* + 04	−2.346*E* + 04	−1.761*E* + 04	−2.955*E* + 04	−2.184*E* + 04	**−3.456E + 04**
	Ave	−1.050*E* + 04	−6.759*E* + 03	−1.623*E* + 04	−3.407*E* + 04	−3.358*E* + 04	−3.150*E* + 04	−2.276*E* + 04	−3.604*E* + 04	−3.379*E* + 04	**−4.050E + 04**
	Std	3.860*E* + 03	**4.827E + 02**	2.907*E* + 03	5.642*E* + 03	5.637*E* + 03	6.075*E* + 03	2.130*E* + 03	4.310*E* + 03	7.876*E* + 03	2.173*E* + 03
	Rank	9	10	8	3	5	6	7	2	4	1
F9	Best	4.779*E* + 02	5.265*E* + 01	2.724*E* − 09	**0**	**0**	**0**	**0**	**0**	**0**	**0**
	Worst	7.998*E* + 02	4.466*E* + 02	4.533*E* + 01	**0**	1.137*E* − 13	**0**	**0**	**0**	**0**	**0**
	Ave	6.303*E* + 02	2.595*E* + 02	3.979*E* + 00	**0**	3.790*E* − 15	**0**	**0**	**0**	**0**	**0**
	Std	8.286*E* + 01	1.086*E* + 02	8.599*E* + 00	**0**	2.076*E* − 14	**0**	**0**	**0**	**0**	**0**
	Rank	10	9	8	1	7	1	1	1	1	1
F10	Best	3.132*E* + 00	9.221*E* + 00	3.430*E* − 07	**8.882E − 16**	**8.882E − 16**	**8.882E − 16**	**8.882E − 16**	**8.882E − 16**	**8.882E − 16**	**8.882E − 16**
	Worst	4.305*E* + 00	2.067*E* + 01	1.375*E* − 06	7.994*E* − 15	7.994*E* − 15	**8.882E − 16**	**8.882E − 16**	**8.882E − 16**	**8.882E − 16**	**8.882E − 16**
	Ave	3.726*E* + 00	1.899*E* + 01	6.452*E* − 07	4.322*E* − 15	4.204*E* − 15	**0**	**0**	**0**	**0**	**0**
	Std	2.946*E* − 01	3.752*E* + 00	2.326*E* − 07	2.873*E* − 15	2.273*E* − 15	**4.012E − 31**	**4.012E − 31**	**4.012E − 31**	**4.012E − 31**	**4.012E − 31**
	Rank	9	10	8	7	6	1	1	1	1	1
F11	Best	2.761*E* − 01	9.224*E* + 00	4.273*E* − 12	**0**	**0**	**0**	**0**	**0**	**0**	**0**
	Worst	6.430*E* − 01	1.889*E* + 02	2.807*E* − 02	3.137*E* − 01	**0**	**0**	**0**	**0**	**0**	**0**
	Ave	4.252*E* − 01	8.541*E* + 01	2.511*E* − 03	1.046*E* − 02	**0**	**0**	**0**	**0**	**0**	**0**
	Std	9.838*E* − 02	5.334*E* + 01	7.694*E* − 03	5.728*E* − 02	**0**	**0**	**0**	**0**	**0**	**0**
	Rank	9	10	7	8	1	1	1	1	1	1
F12	Best	9.845*E* − 01	7.795*E* + 07	1.679*E* − 01	1.867*E* − 02	1.876*E* − 02	2.330*E* − 20	1.123*E* − 12	2.531*E* − 12	3.574*E* − 13	**4.712E − 33**
	Worst	9.589*E* + 00	6.179*E* + 08	3.925*E* − 01	9.377*E* − 02	1.440*E* − 01	**8.878E − 09**	6.918*E* − 08	1.562*E* − 07	1.101*E* − 08	9.480*E* − 05
	Ave	4.297*E* + 00	2.889*E* + 08	2.624*E* − 01	4.903*E* − 02	4.792*E* − 02	**9.451E − 10**	1.021*E* − 08	1.261*E* − 08	2.028*E* − 09	2.722*E* − 05
	Std	1.884*E* + 00	1.390*E* + 08	6.522*E* − 02	1.946*E* − 02	2.491*E* − 02	**1.909E − 09**	1.627*E* − 08	2.920*E* − 08	2.960*E* − 09	3.211*E* − 05
	Rank	9	10	8	7	6	1	3	4	2	5
F13	Best	2.737*E* + 01	2.004*E* + 08	5.195*E* + 00	1.224*E* + 00	1.242*E* + 00	6.058*E* − 14	7.614*E* − 10	6.218*E* − 10	1.775*E* − 10	**1.350E − 32**
	Worst	9.778*E* + 01	1.593*E* + 09	6.920*E* + 00	5.151*E* + 00	5.166*E* + 00	4.220*E* − 07	3.989*E* − 06	1.629*E* − 06	6.166*E* − 06	**1.350E − 32**
	Ave	5.972*E* + 01	6.758*E* + 08	6.397*E* + 00	2.787*E* + 00	3.138*E* + 00	4.654*E* − 08	4.157*E* − 07	2.250*E* − 07	5.104*E* − 07	**1.350E − 32**
	Std	1.998*E* + 01	3.405*E* + 08	4.285*E* − 01	9.742*E* − 01	8.932*E* − 01	9.147*E* − 08	8.210*E* − 07	4.018*E* − 07	1.269*E* − 06	**0**
	Rank	9	10	8	6	7	2	4	3	5	1
Average rank		8.92	9.62	7.46	6.38	6.15	3.31	3.46	1.70	2.85	1.61

**Table 7:** Wilcoxon rank sum test results of each algorithm (30 dimensions).

*F*	PSO	SCA	GWO	WOA	MWOA	SSA	BSSA	CSSA	LSSA
F1	1.212*E* − 12	1.212*E* − 12	1.212*E* − 12	1.212*E* − 12	1.212*E* − 12	NaN	1.212*E* − 12	NaN	NaN
F2	1.212*E* − 12	1.212*E* − 12	1.212*E* − 12	1.212*E* − 12	1.212*E* − 12	4.057*E* − 03	1.212*E* − 12	NaN	1.212*E* − 12
F3	1.212*E* − 12	1.212*E* − 12	1.212*E* − 12	1.212*E* − 12	1.212*E* − 12	5.772*E* − 11	1.212*E* − 12	NaN	4.714*E* − 05
F4	1.212*E* − 12	1.212*E* − 12	1.212*E* − 12	1.212*E* − 12	1.212*E* − 12	1.212*E* − 12	1.212*E* − 12	4.574*E* − 12	1.212*E* − 12
F5	1.212*E* − 12	1.212*E* − 12	1.212*E* − 12	1.212*E* − 12	1.211*E* − 12	1.212*E* − 12	1.212*E* − 12	1.212*E* − 12	1.212*E* − 12
F6	1.167*E* − 02	2.954*E* − 11	2.814*E* − 10	2.954*E* − 11	2.954*E* − 11	1.957*E* − 04	3.962*E* − 04	3.540*E* − 04	3.962*E* − 04
F7	3.020*E* − 11	3.020*E* − 11	4.504*E* − 11	9.063*E* − 08	4.616*E* − 10	1.023*E* − 01	9.626*E* − 02	7.062*E* − 01	7.483*E* − 02
F8	2.392*E* − 11	2.392*E* − 11	2.392*E* − 11	2.831*E* − 07	3.102*E* − 08	1.783*E* − 06	2.392*E* − 11	3.327*E* − 09	4.347*E* − 08
F9	1.212*E* − 12	1.212*E* − 12	3.818*E* − 12	NaN	3.337*E* − 01	NaN	NaN	NaN	NaN
F10	1.211*E* − 12	1.212*E* − 12	1.199*E* − 12	9.318*E* − 08	1.317*E* − 09	NaN	NaN	NaN	NaN
F11	1.212*E* − 12	1.212*E* − 12	6.617*E* − 04	3.337*E* − 01	NaN	NaN	NaN	NaN	NaN
F12	1.259*E* − 01	2.982*E* − 11	2.982*E* − 11	2.982*E* − 11	2.982*E* − 11	9.460*E* − 06	9.460*E* − 06	9.460*E* − 06	2.420*E* − 05
F13	1.211*E* − 12	1.212*E* − 12	1.212*E* − 12	1.212*E* − 12	1.212*E* − 12	1.212*E* − 12	1.212*E* − 12	1.212*E* − 12	1.212*E* − 12
F14	4.126*E* − 07	3.800*E* − 09	1.642*E* − 05	2.328*E* − 04	3.743*E* − 05	5.429*E* − 08	1.058*E* − 10	1.608*E* − 01	4.181*E* − 01
F15	5.992*E* − 11	2.982*E* − 11	8.620*E* − 09	1.081*E* − 10	4.027*E* − 11	9.080*E* − 03	1.598*E* − 01	3.241*E* − 06	3.455*E* − 03
F16	1.685*E* − 14	3.620*E* − 13	1.685*E* − 14	1.685*E* − 14	1.685*E* − 14	2.708*E* − 14	1.685*E* − 14	1.685*E* − 14	1.685*E* − 14
F17	NaN	4.566*E* − 12	3.337*E* − 01	6.519*E* − 04	6.500*E* − 05	NaN	NaN	NaN	NaN
F18	NaN	NaN	NaN	NaN	NaN	1.607*E* − 01	NaN	NaN	NaN
F19	NaN	1.129*E* − 12	2.158*E* − 02	1.828*E* − 09	5.600*E* − 10	NaN	NaN	NaN	NaN
F20	6.138*E* − 01	6.738*E* − 11	2.457*E* − 02	1.021*E* − 01	4.245*E* − 04	1.000*E* + 00	1.189*E* − 01	5.271*E* − 05	3.055*E* − 01
F21	1.173*E* − 05	1.212*E* − 12	1.101*E* − 02	1.878*E* − 09	5.404*E* − 11	NaN	NaN	NaN	NaN
F22	4.721*E* − 03	1.720*E* − 12	1.000*E* + 00	1.704*E* − 12	1.715*E* − 12	2.416*E* − 04	1.039*E* − 10	3.337*E* − 01	3.337*E* − 01
F23	2.157*E* − 02	1.212*E* − 12	1.608*E* − 01	1.208*E* − 12	1.208*E* − 12	2.773*E* − 03	5.808*E* − 09	NaN	NaN

**Table 8:** Test results of each algorithm in CEC 2017.

F	Index	PSO	SCA	GWO	WOA	MWOA	SSA	BSSA	CSSA	LSSA	ISSA
F1	Best	1.52*E* + 02	9.06*E* + 09	5.10*E* + 08	4.33*E* + 06	4.28*E* + 09	1.09*E* + 02	1.65*E* + 07	1.23*E* + 02	1.06*E* + 02	1.00*E* + 02
	Worst	9.74*E* + 03	1.84*E* + 10	3.22*E* + 09	1.17*E* + 08	1.04*E* + 10	1.99*E* + 04	1.66*E* + 08	1.20*E* + 04	2.08*E* + 04	**9.24E + 03**
	Med	1.68*E* + 03	1.19*E* + 10	1.05*E* + 09	2.06*E* + 07	6.36*E* + 09	6.79*E* + 02	8.20*E* + 07	1.48*E* + 03	5.36*E* + 03	**5.23E + 02**
	Ave	2.53*E* + 03	1.20*E* + 10	1.35*E* + 09	2.99*E* + 07	6.43*E* + 09	3.47*E* + 03	9.87*E* + 07	3.18*E* + 03	8.01*E* + 03	**1.78E + 03**
	Std	2.61*E* + 03	2.06*E* + 09	7.53*E* + 08	2.74*E* + 07	1.21*E* + 09	5.82*E* + 03	4.14*E* + 07	3.29*E* + 03	7.96*E* + 03	**2.32E + 03**
	Rank	2	10	8	6	9	4	7	3	5	1
F3	Best	1.95*E* + 03	2.30*E* + 04	2.87*E* + 04	8.52*E* + 04	4.82*E* + 04	1.26*E* + 04	4.67*E* + 04	5.46*E* + 03	2.15*E* + 04	**3.07E + 02**
	Worst	1.19*E* + 04	4.89*E* + 04	6.27*E* + 04	3.02*E* + 05	6.49*E* + 04	2.98*E* + 04	6.87*E* + 04	1.44*E* + 04	4.99*E* + 04	**7.66E + 02**
	Med	5.12*E* + 03	3.46*E* + 04	5.06*E* + 04	1.89*E* + 05	5.56*E* + 04	1.67*E* + 04	5.67*E* + 04	1.02*E* + 04	3.30*E* + 04	**4.90E + 02**
	Ave	5.49*E* + 03	3.39*E* + 04	4.92*E* + 04	2.00*E* + 05	5.53*E* + 04	1.78*E* + 04	5.69*E* + 04	9.94*E* + 03	3.21*E* + 04	**4.64E + 02**
	Std	1.58*E* + 03	8.48*E* + 03	1.15*E* + 04	7.55*E* + 04	3.44*E* + 03	4.49*E* + 03	8.03*E* + 03	2.35*E* + 03	7.20*E* + 03	**1.20E + 02**
	Rank	2	6	7	10	8	4	9	3	5	1
F4	Best	4.18*E* + 02	8.86*E* + 02	5.20*E* + 02	5.03*E* + 02	1.23*E* + 03	4.59*E* + 02	5.10*E* + 02	4.59*E* + 02	4.08*E* + 02	**4.04E + 02**
	Worst	**4.82E + 02**	1.89*E* + 03	6.49*E* + 02	6.48*E* + 02	2.73*E* + 03	5.38*E* + 02	6.93*E* + 02	5.35*E* + 02	5.40*E* + 02	6.09*E* + 02
	Med	4.79*E* + 02	1.53*E* + 03	6.18*E* + 02	5.58*E* + 02	2.13*E* + 03	5.10*E* + 02	5.80*E* + 02	4.88*E* + 02	**4.72E + 02**	4.77*E* + 02
	Ave	**4.63E + 02**	1.48*E* + 03	6.09*E* + 02	5.61*E* + 02	2.08*E* + 03	5.08*E* + 02	5.78*E* + 02	4.83*E* + 02	4.82*E* + 02	4.66*E* + 02
	Std	2.67*E* + 01	2.49*E* + 02	3.67*E* + 01	4.26*E* + 01	4.82*E* + 02	1.79*E* + 01	5.62*E* + 01	**1.56E + 01**	2.91*E* + 01	4.15*E* + 01
	Rank	1	9	8	6	10	5	7	4	3	2
F5	Best	6.31*E* + 02	7.39*E* + 02	**5.53E + 02**	7.05*E* + 02	7.99*E* + 02	7.34*E* + 02	5.91*E* + 02	6.56*E* + 02	6.21*E* + 02	6.49*E* + 02
	Worst	7.30*E* + 02	8.27*E* + 02	7.47*E* + 02	9.26*E* + 02	9.37*E* + 02	8.20*E* + 02	**6.70E + 02**	8.07*E* + 02	8.21*E* + 02	8.16*E* + 02
	Med	7.00*E* + 02	7.62*E* + 02	**5.94E + 02**	7.57*E* + 02	8.49*E* + 02	8.15*E* + 02	6.45*E* + 02	7.40*E* + 02	7.18*E* + 02	7.80*E* + 02
	Ave	6.90*E* + 02	7.68*E* + 02	**6.03E + 02**	7.80*E* + 02	8.69*E* + 02	8.08*E* + 02	6.34*E* + 02	7.40*E* + 02	7.32*E* + 02	7.62*E* + 02
	Std	3.06*E* + 01	**1.73E + 01**	3.50*E* + 01	7.36*E* + 01	4.91*E* + 01	1.79*E* + 01	2.40*E* + 01	4.57*E* + 01	5.45*E* + 01	3.41*E* + 01
	Rank	3	7	1	8	10	9	2	5	4	6
F6	Best	6.34*E* + 02	6.42*E* + 02	6.38*E* + 02	6.61*E* + 02	6.65*E* + 02	6.57*E* + 02	6.08*E* + 02	6.21*E* + 02	6.22*E* + 02	**6.02E + 02**
	Worst	6.57*E* + 02	6.61*E* + 02	6.68*E* + 02	6.83*E* + 02	6.88*E* + 02	6.78*E* + 02	6.65*E* + 02	6.57*E* + 02	6.58*E* + 02	**6.12E + 02**
	Med	6.45*E* + 02	6.50*E* + 02	6.57*E* + 02	6.70*E* + 02	6.76*E* + 02	6.64*E* + 02	6.29*E* + 02	6.27*E* + 02	6.41*E* + 02	**6.11E + 02**
	Ave	6.46*E* + 02	6.49*E* + 02	6.56*E* + 02	6.72*E* + 02	6.74*E* + 02	6.64*E* + 02	6.38*E* + 02	6.33*E* + 02	6.41*E* + 02	**6.08E + 02**
	Std	7.80*E* + 00	5.44*E* + 00	6.90*E* + 00	8.86*E* + 00	5.46*E* + 00	6.39*E* + 00	2.16*E* + 01	1.08*E* + 01	1.02*E* + 01	**4.37E + 00**
	Rank	5	6	7	9	10	8	3	2	4	1
F7	Best	1.07*E* + 03	1.08*E* + 03	8.20*E* + 02	1.06*E* + 03	1.19*E* + 03	1.18*E* + 03	8.74*E* + 02	9.28*E* + 02	8.64*E* + 02	**7.99E + 02**
	Worst	1.37*E* + 03	1.18*E* + 03	9.91*E* + 02	1.44*E* + 03	1.41*E* + 03	1.35*E* + 03	1.33*E* + 03	1.34*E* + 03	1.33*E* + 03	**8.76E + 02**
	Med	1.32*E* + 03	1.10*E* + 03	8.91*E* + 02	1.24*E* + 03	1.27*E* + 03	1.33*E* + 03	9.82*E* + 02	1.17*E* + 03	1.14*E* + 03	**8.51E + 02**
	Ave	1.29*E* + 03	1.11*E* + 03	8.89*E* + 02	1.28*E* + 03	1.29*E* + 03	1.32*E* + 03	1.00*E* + 03	1.17*E* + 03	1.14*E* + 03	**8.46E + 02**
	Std	6.72*E* + 01	2.99*E* + 01	4.61*E* + 01	1.15*E* + 02	7.20*E* + 01	3.85*E* + 01	8.42*E* + 01	1.09*E* + 02	1.44*E* + 02	**1.66E + 01**
	Rank	8	4	2	7	9	10	3	6	5	1
F8	Best	8.74*E* + 02	1.02*E* + 03	9.08*E* + 02	9.42*E* + 02	1.04*E* + 03	9.38*E* + 02	9.15*E* + 02	8.60*E* + 02	8.84*E* + 02	**8.55E + 02**
	Worst	9.62*E* + 02	1.09*E* + 03	1.01*E* + 03	1.08*E* + 03	1.11*E* + 03	1.05*E* + 03	9.79*E* + 02	9.82*E* + 02	1.01*E* + 03	**9.14E + 02**
	Med	9.15*E* + 02	1.04*E* + 03	9.52*E* + 02	9.72*E* + 02	1.09*E* + 03	9.92*E* + 02	9.59*E* + 02	9.43*E* + 02	9.66*E* + 02	**8.80E + 02**
	Ave	9.21*E* + 02	1.05*E* + 03	9.57*E* + 02	9.89*E* + 02	1.08*E* + 03	9.94*E* + 02	9.53*E* + 02	9.48*E* + 02	9.58*E* + 02	**8.82E + 02**
	Std	2.58*E* + 01	2.28*E* + 01	2.31*E* + 01	3.69*E* + 01	1.63*E* + 01	2.56*E* + 01	1.44*E* + 01	2.79*E* + 01	3.98*E* + 01	**1.37E + 01**
	Rank	2	9	5	7	10	8	4	3	6	1
F9	Best	2.45*E* + 03	3.74*E* + 03	3.57*E* + 03	3.60*E* + 03	6.34*E* + 03	4.01*E* + 03	2.04*E* + 03	3.08*E* + 03	5.06*E* + 03	**1.06E + 03**
	Worst	5.01*E* + 03	7.85*E* + 03	5.79*E* + 03	1.35*E* + 04	1.15*E* + 04	5.58*E* + 03	9.94*E* + 03	5.44*E* + 03	5.56*E* + 03	**1.85E + 03**
	Med	3.99*E* + 03	4.43*E* + 03	4.97*E* + 03	6.12*E* + 03	8.81*E* + 03	5.41*E* + 03	5.85*E* + 03	5.35*E* + 03	5.39*E* + 03	**1.44E + 03**
	Ave	3.84*E* + 03	4.95*E* + 03	4.95*E* + 03	6.82*E* + 03	8.73*E* + 03	5.30*E* + 03	6.02*E* + 03	4.88*E* + 03	5.37*E* + 03	**1.42E + 03**
	Std	5.34*E* + 02	1.09*E* + 03	4.57*E* + 02	1.82*E* + 03	1.34*E* + 03	3.75*E* + 02	2.32*E* + 03	8.19*E* + 02	**1.23E + 02**	1.87*E* + 02
	Rank	2	4	5	9	10	6	8	3	7	1
F10	Best	3.71*E* + 03	7.33*E* + 03	**3.15E + 03**	5.34*E* + 03	5.74*E* + 03	4.57*E* + 03	4.82*E* + 03	4.09*E* + 03	3.91*E* + 03	4.06*E* + 03
	Worst	5.45*E* + 03	8.48*E* + 03	**5.06E + 03**	8.13*E* + 03	8.59*E* + 03	9.10*E* + 03	9.80*E* + 03	6.33*E* + 03	6.53*E* + 03	8.89*E* + 03
	Med	4.27*E* + 03	8.17*E* + 03	**4.20E + 03**	7.29*E* + 03	7.34*E* + 03	5.95*E* + 03	6.57*E* + 03	5.39*E* + 03	4.58*E* + 03	6.24*E* + 03
	Ave	4.47*E* + 03	8.13*E* + 03	**4.05E + 03**	7.00*E* + 03	7.54*E* + 03	6.43*E* + 03	6.71*E* + 03	5.42*E* + 03	4.83*E* + 03	6.09*E* + 03
	Std	5.13*E* + 02	**2.79E + 02**	6.38*E* + 02	9.77*E* + 02	6.40*E* + 02	1.63*E* + 03	1.60*E* + 03	5.13*E* + 02	6.99*E* + 02	1.09*E* + 03
	Rank	2	10	1	8	9	6	7	4	3	5
F11	Best	1.16*E* + 03	1.71*E* + 03	1.34*E* + 03	1.47*E* + 03	2.57*E* + 03	1.19*E* + 03	1.31*E* + 03	1.16*E* + 03	1.25*E* + 03	**1.14E + 03**
	Worst	**1.25E + 03**	3.73*E* + 03	2.12*E* + 03	2.49*E* + 03	5.28*E* + 03	1.39*E* + 03	1.88*E* + 03	1.32*E* + 03	1.59*E* + 03	1.29*E* + 03
	Med	1.19*E* + 03	1.90*E* + 03	1.44*E* + 03	2.30*E* + 03	4.37*E* + 03	1.23*E* + 03	1.43*E* + 03	1.26*E* + 03	1.44*E* + 03	**1.19E + 03**
	Ave	**1.19E + 03**	2.02*E* + 03	1.59*E* + 03	2.12*E* + 03	4.23*E* + 03	1.25*E* + 03	1.45*E* + 03	1.25*E* + 03	1.43*E* + 03	1.19*E* + 03
	Std	**2.46E + 01**	4.01*E* + 02	2.58*E* + 02	3.84*E* + 02	6.64*E* + 02	4.68*E* + 01	1.36*E* + 02	4.06*E* + 01	9.29*E* + 01	3.12*E* + 01
	Rank	1	8	7	9	10	3	6	4	5	2
F12	Best	**2.20E + 04**	6.42*E* + 08	4.00*E* + 06	1.03*E* + 07	6.90*E* + 08	3.78*E* + 04	9.49*E* + 05	1.09*E* + 05	1.25*E* + 05	2.90*E* + 04
	Worst	1.25*E* + 06	1.99*E* + 09	3.22*E* + 08	1.08*E* + 08	2.59*E* + 09	1.13*E* + 07	1.22*E* + 07	**6.57E + 05**	9.09*E* + 05	1.91*E* + 07
	Med	3.54*E* + 05	8.82*E* + 08	3.93*E* + 07	6.62*E* + 07	9.19*E* + 08	2.07*E* + 06	2.91*E* + 06	**2.08E + 05**	4.45*E* + 05	1.54*E* + 06
	Ave	4.22*E* + 05	1.03*E* + 09	6.63*E* + 07	6.01*E* + 07	1.11*E* + 09	2.45*E* + 06	4.41*E* + 06	**2.23E + 05**	4.57*E* + 05	2.11*E* + 06
	Std	3.55*E* + 05	4.03*E* + 08	8.44*E* + 07	2.84*E* + 07	5.40*E* + 08	2.66*E* + 06	3.09*E* + 06	**1.11E + 05**	2.80*E* + 05	3.55*E* + 06
	Rank	2	9	8	7	10	5	6	1	3	4
F13	Best	2.90*E* + 03	2.13*E* + 08	3.83*E* + 04	3.85*E* + 04	6.22*E* + 07	3.05*E* + 03	8.09*E* + 03	2.97*E* + 03	5.95*E* + 03	**1.68E + 03**
	Worst	5.62*E* + 04	6.13*E* + 08	1.40*E* + 08	2.82*E* + 05	4.32*E* + 08	7.25*E* + 04	4.51*E* + 06	7.12*E* + 04	5.45*E* + 04	**1.96E + 04**
	Med	**8.39E + 03**	3.48*E* + 08	1.84*E* + 05	1.22*E* + 05	1.53*E* + 08	1.39*E* + 04	2.34*E* + 04	9.69*E* + 03	2.75*E* + 04	9.36*E* + 03
	Ave	1.21*E* + 04	3.69*E* + 08	3.90*E* + 07	1.19*E* + 05	1.63*E* + 08	2.56*E* + 04	5.41*E* + 05	1.38*E* + 04	2.92*E* + 04	**9.11E + 03**
	Std	1.07*E* + 04	9.87*E* + 07	5.71*E* + 07	5.05*E* + 04	8.14*E* + 07	2.50*E* + 04	1.36*E* + 06	1.58*E* + 04	2.09*E* + 04	**4.37E + 03**
	Rank	2	10	8	6	9	4	7	3	5	1
F14	Best	4.96*E* + 03	4.08*E* + 04	2.93*E* + 03	1.37*E* + 04	1.41*E* + 06	5.09*E* + 03	7.46*E* + 03	1.67*E* + 03	2.50*E* + 03	**1.66E + 03**
	Worst	1.12*E* + 05	2.71*E* + 05	8.84*E* + 05	7.05*E* + 06	4.40*E* + 06	1.64*E* + 05	1.79*E* + 06	7.66*E* + 04	1.09*E* + 05	**5.18E + 04**
	Med	2.71*E* + 04	8.97*E* + 04	4.63*E* + 04	1.11*E* + 06	2.68*E* + 06	3.07*E* + 04	3.35*E* + 05	1.39*E* + 04	2.86*E* + 04	**5.97E + 03**
	Ave	3.93*E* + 04	1.25*E* + 05	2.31*E* + 05	1.86*E* + 06	2.54*E* + 06	3.37*E* + 04	4.24*E* + 05	1.69*E* + 04	3.86*E* + 04	**1.52E + 04**
	Std	2.72*E* + 04	6.22*E* + 04	2.90*E* + 05	1.72*E* + 06	9.81*E* + 05	2.81*E* + 04	4.18*E* + 05	1.72*E* + 04	3.18*E* + 04	**1.71E + 04**
	Rank	5	6	7	9	10	3	8	2	4	1
F15	Best	1.65*E* + 03	6.16*E* + 05	1.39*E* + 04	2.15*E* + 04	3.22*E* + 06	2.08*E* + 03	2.30*E* + 03	1.85*E* + 03	1.86*E* + 03	**1.57E + 03**
	Worst	2.12*E* + 04	5.07*E* + 07	5.42*E* + 05	1.83*E* + 05	5.30*E* + 07	2.29*E* + 04	2.04*E* + 04	**1.50E + 04**	4.43*E* + 04	3.46*E* + 04
	Med	4.05*E* + 03	5.21*E* + 06	1.03*E* + 05	4.40*E* + 04	7.88*E* + 06	5.81*E* + 03	6.81*E* + 03	3.46*E* + 03	2.92*E* + 04	**2.38E + 03**
	Ave	5.39*E* + 03	1.11*E* + 07	1.12*E* + 05	6.36*E* + 04	1.36*E* + 07	7.88*E* + 03	6.92*E* + 03	**4.75E + 03**	2.35*E* + 04	4.92*E* + 03
	Std	5.43*E* + 03	1.08*E* + 07	1.23*E* + 05	4.40*E* + 04	1.44*E* + 07	6.35*E* + 03	3.96*E* + 03	**3.14E + 03**	1.80*E* + 04	6.67*E* + 03
	Rank	3	9	8	7	10	5	4	1	6	2
F16	Best	2.24*E* + 03	3.37*E* + 03	1.98*E* + 03	2.93*E* + 03	3.86*E* + 03	2.83*E* + 03	2.19*E* + 03	2.42*E* + 03	2.33*E* + 03	**1.88E + 03**
	Worst	**3.06E + 03**	4.09*E* + 03	3.12*E* + 03	4.56*E* + 03	5.69*E* + 03	8.55*E* + 03	3.60*E* + 03	3.30*E* + 03	3.33*E* + 03	5.57*E* + 03
	Med	2.85*E* + 03	3.66*E* + 03	**2.77E + 03**	3.33*E* + 03	4.15*E* + 03	3.79*E* + 03	2.81*E* + 03	3.10*E* + 03	2.89*E* + 03	3.23*E* + 03
	Ave	2.80*E* + 03	3.68*E* + 03	**2.56E + 03**	3.44*E* + 03	4.31*E* + 03	4.12*E* + 03	2.84*E* + 03	3.00*E* + 03	2.87*E* + 03	3.35*E* + 03
	Std	2.70*E* + 02	**1.55E + 02**	4.02*E* + 02	4.03*E* + 02	4.33*E* + 02	1.23*E* + 03	3.20*E* + 02	2.71*E* + 02	2.51*E* + 02	1.09*E* + 03
	Rank	2	8	1	7	10	9	3	5	4	6
F17	Best	1.94*E* + 03	2.11*E* + 03	2.07*E* + 03	2.15*E* + 03	2.22*E* + 03	1.99*E* + 03	1.96*E* + 03	2.05*E* + 03	2.13*E* + 03	**1.83E + 03**
	Worst	2.75*E* + 03	2.69*E* + 03	3.81*E* + 03	3.22*E* + 03	3.15*E* + 03	3.36*E* + 03	2.92*E* + 03	3.01*E* + 03	3.02*E* + 03	**2.25E + 03**
	Med	2.32*E* + 03	2.30*E* + 03	3.08*E* + 03	2.56*E* + 03	2.95*E* + 03	2.68*E* + 03	2.40*E* + 03	2.44*E* + 03	2.65*E* + 03	**1.99E + 03**
	Ave	2.33*E* + 03	2.37*E* + 03	3.00*E* + 03	2.65*E* + 03	2.89*E* + 03	2.81*E* + 03	2.42*E* + 03	2.46*E* + 03	2.58*E* + 03	**2.01E + 03**
	Std	2.55*E* + 02	**1.44E + 02**	4.67*E* + 02	2.65*E* + 02	2.49*E* + 02	3.40*E* + 02	2.45*E* + 02	2.11*E* + 02	2.25*E* + 02	1.48*E* + 02
	Rank	2	3	10	7	9	8	4	5	6	1
F18	Best	5.23*E* + 04	3.20*E* + 05	4.38*E* + 04	2.75*E* + 05	2.86*E* + 06	1.19*E* + 04	5.88*E* + 04	3.78*E* + 04	1.09*E* + 05	**6.74E + 03**
	Worst	5.93*E* + 05	6.66*E* + 06	1.06*E* + 07	8.14*E* + 06	2.41*E* + 07	1.54*E* + 06	7.28*E* + 06	4.14*E* + 05	1.47*E* + 06	**1.74E + 05**
	Med	3.46*E* + 05	1.48*E* + 06	4.59*E* + 05	2.96*E* + 06	8.73*E* + 06	7.18*E* + 04	5.67*E* + 05	8.42*E* + 04	2.53*E* + 05	**4.00E + 04**
	Ave	2.74*E* + 05	1.83*E* + 06	1.55*E* + 06	2.81*E* + 06	8.77*E* + 06	1.80*E* + 05	1.79*E* + 06	1.43*E* + 05	3.56*E* + 05	**7.38E + 04**
	Std	1.84*E* + 05	1.65*E* + 06	2.78*E* + 06	2.01*E* + 06	3.77*E* + 06	3.80*E* + 05	2.81*E* + 06	1.22*E* + 05	3.37*E* + 05	**6.25E + 04**
	Rank	4	8	6	9	10	3	7	2	5	1
F19	Best	2.04*E* + 03	4.15*E* + 06	2.06*E* + 04	9.08*E* + 05	6.49*E* + 06	2.53*E* + 03	2.57*E* + 03	2.17*E* + 03	2.36*E* + 03	**2.02E + 03**
	Worst	2.18*E* + 04	3.99*E* + 07	4.22*E* + 06	1.30*E* + 07	6.12*E* + 07	1.63*E* + 04	2.10*E* + 04	**9.56E + 03**	5.64*E* + 04	2.64*E* + 04
	Med	**3.82E + 03**	2.04*E* + 07	5.12*E* + 05	3.18*E* + 06	1.39*E* + 07	5.51*E* + 03	4.44*E* + 03	5.15*E* + 03	6.78*E* + 03	6.49*E* + 03
	Ave	**5.54E + 03**	2.38*E* + 07	9.64*E* + 05	4.52*E* + 06	2.01*E* + 07	6.91*E* + 03	7.75*E* + 03	5.63*E* + 03	1.09*E* + 04	7.79*E* + 03
	Std	4.49*E* + 03	9.96*E* + 06	1.34*E* + 06	4.12*E* + 06	1.30*E* + 07	3.76*E* + 03	6.79*E* + 03	**2.43E + 03**	1.36*E* + 04	5.90*E* + 03
	Rank	1	10	7	8	9	3	4	2	6	5
F20	Best	2.26*E* + 03	2.38*E* + 03	2.27*E* + 03	2.38*E* + 03	2.49*E* + 03	2.39*E* + 03	**2.14E + 03**	2.29*E* + 03	2.30*E* + 03	2.15*E* + 03
	Worst	2.89*E* + 03	**2.73E + 03**	3.39*E* + 03	3.15*E* + 03	2.99*E* + 03	3.43*E* + 03	3.22*E* + 03	2.95*E* + 03	2.91*E* + 03	2.77*E* + 03
	Med	2.44*E* + 03	2.58*E* + 03	3.04*E* + 03	2.80*E* + 03	2.78*E* + 03	2.75*E* + 03	2.66*E* + 03	2.54*E* + 03	2.71*E* + 03	**2.27E + 03**
	Ave	2.49*E* + 03	2.59*E* + 03	2.97*E* + 03	2.74*E* + 03	2.78*E* + 03	2.84*E* + 03	2.60*E* + 03	2.52*E* + 03	2.69*E* + 03	**2.30E + 03**
	Std	1.63*E* + 02	**8.77E + 01**	2.43*E* + 02	2.01*E* + 02	1.16*E* + 02	2.39*E* + 02	1.92*E* + 02	1.83*E* + 02	1.27*E* + 02	1.30*E* + 02
	Rank	2	4	10	7	8	9	5	3	6	1
F21	Best	2.39*E* + 03	2.52*E* + 03	2.42*E* + 03	2.50*E* + 03	2.56*E* + 03	2.49*E* + 03	2.40*E* + 03	**2.20E + 03**	2.42*E* + 03	2.36*E* + 03
	Worst	2.51*E* + 03	2.60*E* + 03	2.68*E* + 03	2.68*E* + 03	2.74*E* + 03	2.73*E* + 03	2.49*E* + 03	2.58*E* + 03	2.58*E* + 03	**2.46E + 03**
	Med	2.45*E* + 03	2.53*E* + 03	2.54*E* + 03	2.57*E* + 03	2.63*E* + 03	2.62*E* + 03	2.43*E* + 03	2.49*E* + 03	2.46*E* + 03	**2.39E + 03**
	Ave	2.45*E* + 03	2.54*E* + 03	2.53*E* + 03	2.58*E* + 03	2.63*E* + 03	2.59*E* + 03	2.44*E* + 03	2.48*E* + 03	2.48*E* + 03	**2.41E + 03**
	Std	2.32*E* + 01	**1.77E + 01**	5.36*E* + 01	5.01*E* + 01	3.40*E* + 01	5.86*E* + 01	3.25*E* + 01	6.21*E* + 01	4.72*E* + 01	3.81*E* + 01
	Rank	3	7	6	8	10	9	2	4	5	1
F22	Best	**2.30E + 03**	3.96*E* + 03	2.50*E* + 03	2.33*E* + 03	3.59*E* + 03	2.38*E* + 03	2.34*E* + 03	**2.30E + 03**	**2.30E + 03**	2.38*E* + 03
	Worst	**6.81E + 03**	9.97*E* + 03	1.00*E* + 04	8.80*E* + 03	1.03*E* + 04	1.04*E* + 04	8.79*E* + 03	7.87*E* + 03	7.58*E* + 03	9.42*E* + 03
	Med	5.86*E* + 03	9.60*E* + 03	4.99*E* + 03	7.17*E* + 03	4.34*E* + 03	7.44*E* + 03	2.38*E* + 03	**2.30E + 03**	5.73*E* + 03	7.31*E* + 03
	Ave	4.91*E* + 03	9.00*E* + 03	4.48*E* + 03	6.68*E* + 03	6.02*E* + 03	7.56*E* + 03	**3.30E + 03**	3.34*E* + 03	5.46*E* + 03	7.27*E* + 03
	Std	1.90*E* + 03	1.71*E* + 03	1.98*E* + 03	1.76*E* + 03	2.55*E* + 03	1.42*E* + 03	2.12*E* + 03	1.94*E* + 03	2.08*E* + 03	**1.30E + 03**
	Rank	4	10	3	7	6	9	1	2	5	8
F23	Best	**2.70E + 03**	2.95*E* + 03	2.71*E* + 03	2.93*E* + 03	2.99*E* + 03	2.99*E* + 03	2.76*E* + 03	2.83*E* + 03	2.78*E* + 03	2.85*E* + 03
	Worst	3.36*E* + 03	3.05*E* + 03	2.95*E* + 03	3.29*E* + 03	3.25*E* + 03	3.57*E* + 03	**2.91E + 03**	3.06*E* + 03	2.95*E* + 03	3.59*E* + 03
	Med	3.15*E* + 03	2.97*E* + 03	**2.75E + 03**	3.03*E* + 03	3.05*E* + 03	3.33*E* + 03	2.83*E* + 03	2.92*E* + 03	2.82*E* + 03	3.31*E* + 03
	Ave	3.16*E* + 03	2.98*E* + 03	**2.77E + 03**	3.02*E* + 03	3.10*E* + 03	3.33*E* + 03	2.82*E* + 03	2.93*E* + 03	2.83*E* + 03	3.26*E* + 03
	Std	1.45*E* + 02	**3.02E + 01**	5.08*E* + 01	9.83*E* + 01	9.88*E* + 01	1.35*E* + 02	4.96*E* + 01	6.17*E* + 01	4.42*E* + 01	1.70*E* + 02
	Rank	8	5	1	6	7	10	2	4	3	9
F24	Best	3.07*E* + 03	3.13*E* + 03	3.11*E* + 03	3.00*E* + 03	3.13*E* + 03	3.19*E* + 03	2.91*E* + 03	2.98*E* + 03	2.93*E* + 03	**2.87E + 03**
	Worst	3.40*E* + 03	3.20*E* + 03	3.77*E* + 03	3.36*E* + 03	3.31*E* + 03	3.69*E* + 03	**3.06E + 03**	3.37*E* + 03	3.10*E* + 03	3.08*E* + 03
	Med	3.23*E* + 03	3.17*E* + 03	3.34*E* + 03	3.19*E* + 03	3.20*E* + 03	3.34*E* + 03	3.01*E* + 03	3.13*E* + 03	3.01*E* + 03	**2.91E + 03**
	Ave	3.24*E* + 03	3.17*E* + 03	3.40*E* + 03	3.17*E* + 03	3.21*E* + 03	3.33*E* + 03	2.99*E* + 03	3.14*E* + 03	3.01*E* + 03	**2.93E + 03**
	Std	1.33*E* + 02	**1.72E + 01**	1.94*E* + 02	7.64*E* + 01	5.73*E* + 01	1.05*E* + 02	4.18*E* + 01	1.03*E* + 02	4.47*E* + 01	5.27*E* + 01
	Rank	8	5	10	6	7	9	2	4	3	1
F25	Best	2.88*E* + 03	3.11*E* + 03	2.92*E* + 03	2.91*E* + 03	3.30*E* + 03	2.88*E* + 03	2.90*E* + 03	2.88*E* + 03	2.88*E* + 03	**2.88E + 03**
	Worst	2.90*E* + 03	3.36*E* + 03	3.03*E* + 03	3.05*E* + 03	3.55*E* + 03	2.95*E* + 03	3.02*E* + 03	2.94*E* + 03	2.89*E* + 03	2.95*E* + 03
	Med	**2.88E + 03**	3.22*E* + 03	2.98*E* + 03	2.93*E* + 03	3.39*E* + 03	2.90*E* + 03	2.96*E* + 03	2.89*E* + 03	2.89*E* + 03	2.89*E* + 03
	Ave	**2.88E + 03**	3.23*E* + 03	2.98*E* + 03	2.95*E* + 03	3.38*E* + 03	2.91*E* + 03	2.97*E* + 03	2.90*E* + 03	2.89*E* + 03	2.90*E* + 03
	Std	3.67*E* + 00	6.72*E* + 01	3.00*E* + 01	3.94*E* + 01	5.73*E* + 01	2.12*E* + 01	3.70*E* + 01	1.78*E* + 01	1.51*E* + 00	2.86*E* + 01
	Rank	1	9	8	6	10	5	7	3	2	4
F26	Best	5.76*E* + 03	6.49*E* + 03	4.29*E* + 03	6.03*E* + 03	7.32*E* + 03	3.01*E* + 03	3.55*E* + 03	2.90*E* + 03	2.80*E* + 03	**2.80E + 03**
	Worst	8.91*E* + 03	7.27*E* + 03	**5.10E + 03**	9.96*E* + 03	1.04*E* + 04	1.21*E* + 04	7.60*E* + 03	7.55*E* + 03	6.22*E* + 03	1.09*E* + 04
	Med	6.60*E* + 03	6.97*E* + 03	**4.63E + 03**	7.58*E* + 03	8.11*E* + 03	9.02*E* + 03	5.23*E* + 03	5.60*E* + 03	5.13*E* + 03	6.32*E* + 03
	Ave	7.08*E* + 03	6.92*E* + 03	**4.64E + 03**	7.58*E* + 03	8.29*E* + 03	8.76*E* + 03	5.23*E* + 03	5.65*E* + 03	4.67*E* + 03	6.69*E* + 03
	Std	1.06*E* + 03	1.75*E* + 02	**1.51E + 02**	6.53*E* + 02	6.15*E* + 02	1.80*E* + 03	7.03*E* + 02	1.07*E* + 03	1.35*E* + 03	2.08*E* + 03
	Rank	7	6	1	8	9	10	3	4	2	5
F27	Best	**3.17E + 03**	3.34*E* + 03	3.22*E* + 03	3.27*E* + 03	3.38*E* + 03	3.33*E* + 03	3.22*E* + 03	3.23*E* + 03	3.22*E* + 03	3.20*E* + 03
	Worst	3.42*E* + 03	3.49*E* + 03	3.33*E* + 03	3.68*E* + 03	4.00*E* + 03	4.14*E* + 03	3.40*E* + 03	3.41*E* + 03	3.35*E* + 03	**3.20E + 03**
	Med	**3.19E + 03**	3.41*E* + 03	3.25*E* + 03	3.33*E* + 03	3.60*E* + 03	3.80*E* + 03	3.28*E* + 03	3.30*E* + 03	3.23*E* + 03	3.20*E* + 03
	Ave	3.21*E* + 03	3.41*E* + 03	3.26*E* + 03	3.41*E* + 03	3.61*E* + 03	3.71*E* + 03	3.29*E* + 03	3.32*E* + 03	3.25*E* + 03	**3.20E + 03**
	Std	6.02*E* + 01	3.97*E* + 01	2.94*E* + 01	1.39*E* + 02	1.26*E* + 02	2.21*E* + 02	6.50*E* + 01	6.10*E* + 01	4.13*E* + 01	**1.09E − 04**
	Rank	2	7	4	8	9	10	5	6	3	1
F28	Best	3.10*E* + 03	3.65*E* + 03	3.30*E* + 03	3.27*E* + 03	3.66*E* + 03	3.20*E* + 03	3.26*E* + 03	3.10*E* + 03	3.12*E* + 03	**3.10E + 03**
	Worst	3.27*E* + 03	4.12*E* + 03	3.62*E* + 03	3.40*E* + 03	4.26*E* + 03	3.29*E* + 03	3.37*E* + 03	3.26*E* + 03	**3.26E + 03**	3.30*E* + 03
	Med	3.21*E* + 03	3.82*E* + 03	3.41*E* + 03	3.33*E* + 03	3.87*E* + 03	3.22*E* + 03	3.33*E* + 03	**3.11E + 03**	3.21*E* + 03	3.30*E* + 03
	Ave	3.21*E* + 03	3.85*E* + 03	3.40*E* + 03	3.33*E* + 03	3.95*E* + 03	3.22*E* + 03	3.33*E* + 03	**3.15E + 03**	3.22*E* + 03	3.28*E* + 03
	Std	4.84*E* + 01	1.55*E* + 02	7.88*E* + 01	2.67*E* + 01	1.72*E* + 02	**2.45E + 01**	3.22*E* + 01	6.51*E* + 01	3.33*E* + 01	4.67*E* + 01
	Rank	2	9	8	6	10	3	7	1	4	5
F29	Best	3.60*E* + 03	4.24*E* + 03	3.94*E* + 03	4.17*E* + 03	4.73*E* + 03	4.27*E* + 03	3.59*E* + 03	**3.42E + 03**	3.52*E* + 03	3.58*E* + 03
	Worst	4.30*E* + 03	5.05*E* + 03	8.78*E* + 03	5.73*E* + 03	6.02*E* + 03	7.31*E* + 03	4.26*E* + 03	4.82*E* + 03	4.40*E* + 03	**4.06E + 03**
	Med	3.95*E* + 03	4.53*E* + 03	4.92*E* + 03	4.97*E* + 03	5.11*E* + 03	5.24*E* + 03	4.16*E* + 03	4.09*E* + 03	4.05*E* + 03	**3.78E + 03**
	Ave	3.94*E* + 03	4.59*E* + 03	5.22*E* + 03	4.88*E* + 03	5.22*E* + 03	5.27*E* + 03	4.06*E* + 03	4.27*E* + 03	3.97*E* + 03	**3.79E + 03**
	Std	1.63*E* + 02	2.42*E* + 02	9.10*E* + 02	3.98*E* + 02	3.54*E* + 02	6.08*E* + 02	1.77*E* + 02	4.26*E* + 02	2.14*E* + 02	**1.27E + 02**
	Rank	2	6	8	7	9	10	4	5	3	1
F30	Best	5.40*E* + 03	4.89*E* + 07	1.44*E* + 06	4.12*E* + 06	2.32*E* + 07	1.21*E* + 04	1.11*E* + 04	5.82*E* + 03	6.80*E* + 03	**3.61E + 03**
	Worst	**1.07E + 04**	1.15*E* + 08	3.20*E* + 07	4.34*E* + 07	3.98*E* + 08	6.06*E* + 06	3.05*E* + 05	1.88*E* + 04	2.42*E* + 04	1.29*E* + 05
	Med	**6.25E + 03**	7.19*E* + 07	7.15*E* + 06	1.34*E* + 07	1.38*E* + 08	3.41*E* + 04	4.66*E* + 04	1.05*E* + 04	1.97*E* + 04	1.25*E* + 04
	Ave	**6.76E + 03**	7.64*E* + 07	1.60*E* + 07	1.49*E* + 07	1.54*E* + 08	1.25*E* + 06	5.65*E* + 04	1.06*E* + 04	1.72*E* + 04	1.68*E* + 04
	Std	**1.69E + 03**	1.76*E* + 07	1.35*E* + 07	1.22*E* + 07	9.75*E* + 07	2.45*E* + 06	5.64*E* + 04	3.59*E* + 03	6.03*E* + 03	2.37*E* + 04
	Rank	1	9	8	7	10	6	5	2	4	3
Average rank		3.07	7.34	6.00	7.41	9.20	6.66	4.90	3.31	4.35	2.80

**Table 9:** PID parameter setting results.

Algorithm	Unit step				Sinusoidal input			
	Fitness	*K*_*p*_	*K*_*i*_	*K*_*d*_	Fitness	*K*_*p*_	*K*_*i*_	*K*_*d*_
SSA	29.1777	10	0.221046	0.126894	53.0827	10	10	1.90441
ISSA	**22.7045**	41.4593	0.596364	0.402723	**50.5554**	286.6486	21.19153	0.898048

**Table 10:** Robot path planning results.

Algorithm	Fitness			
	Best	Worst	Ave	Std
SSA	22.6274	39.5980	29.9813	6.5115
ISSA	**19.7990**	**28.2843**	**22.6274**	**3.4641**

## Data Availability

The data used to support the findings of this study are available from the corresponding author upon request.
